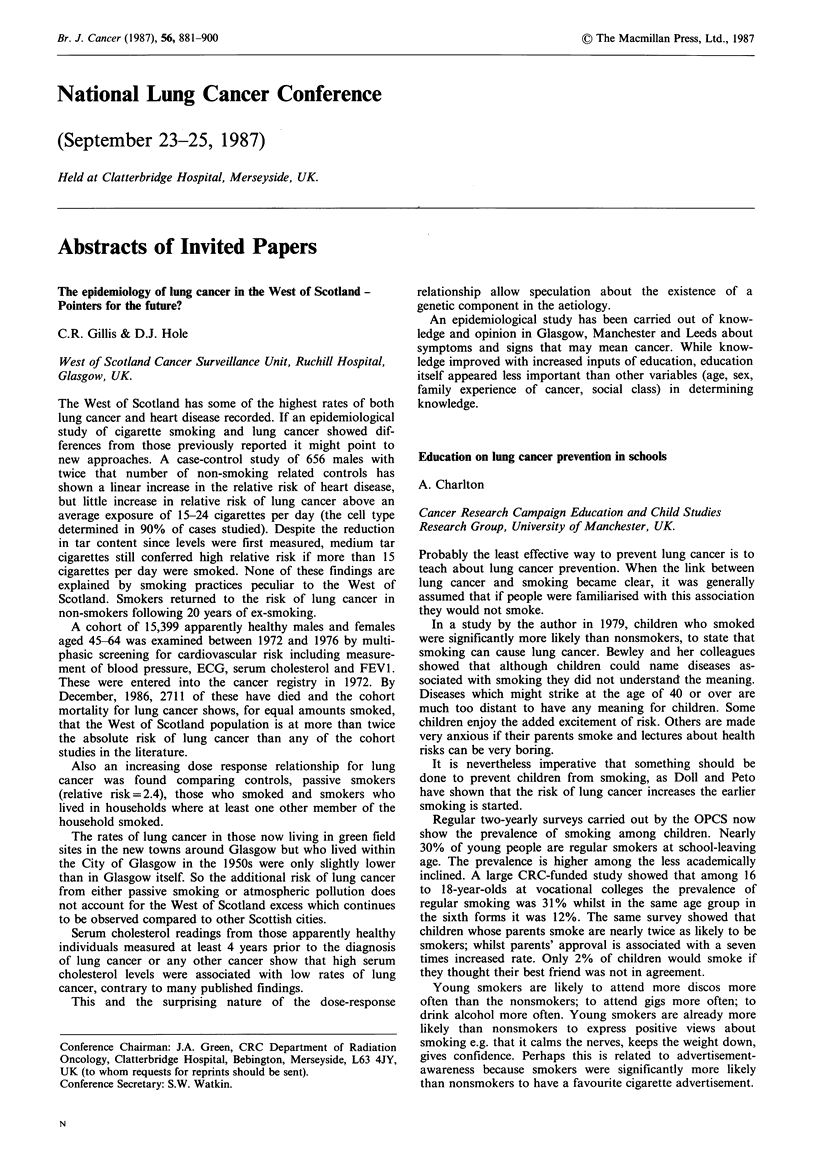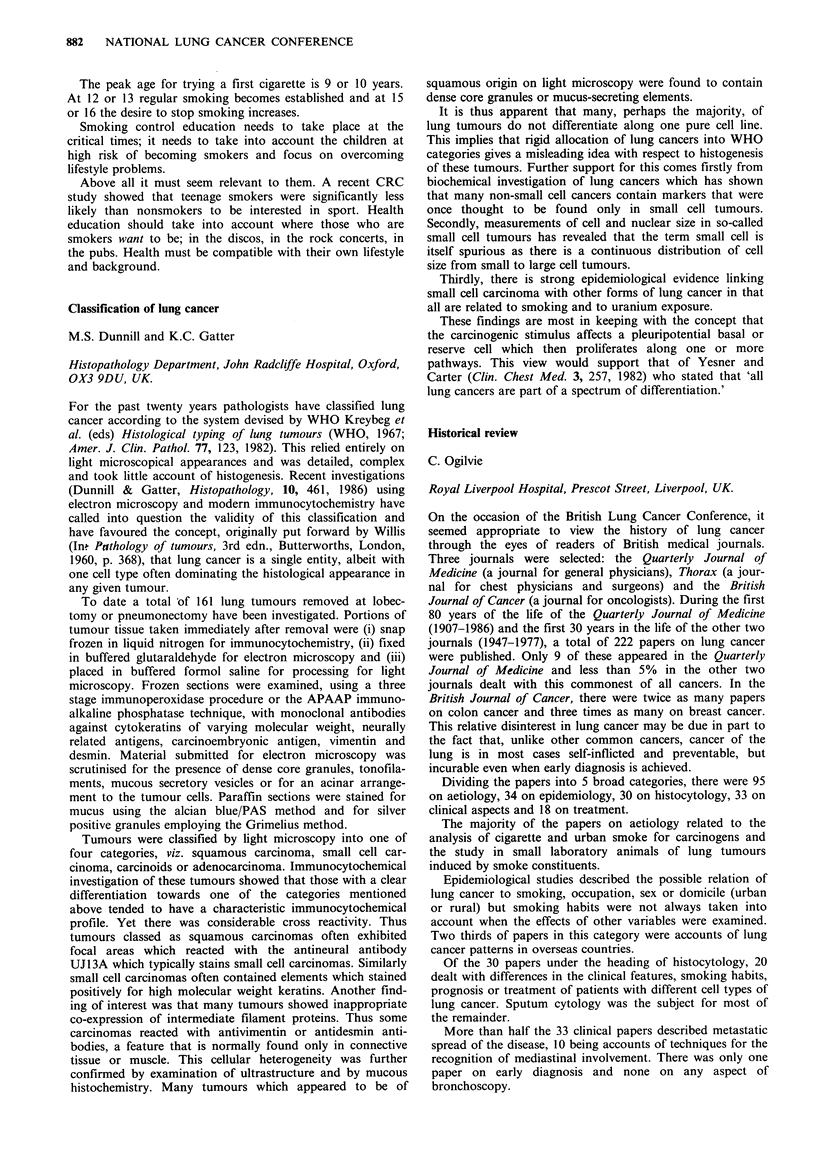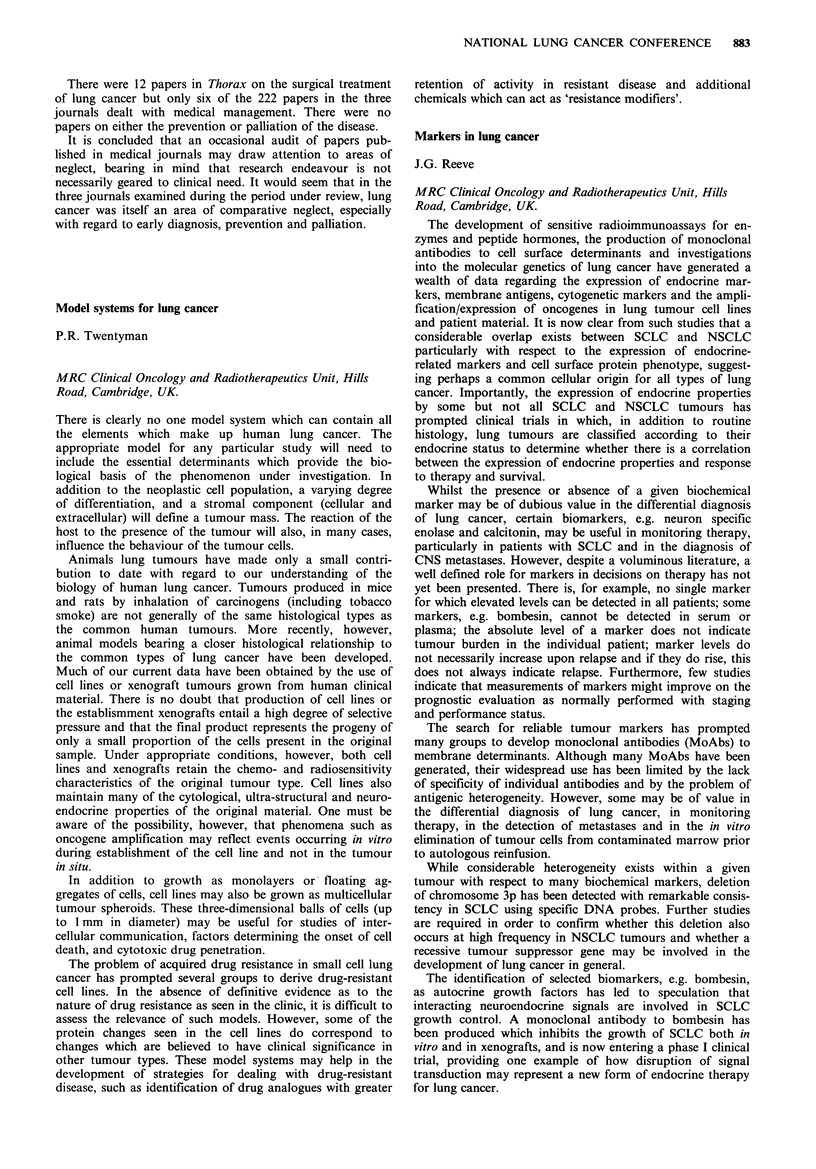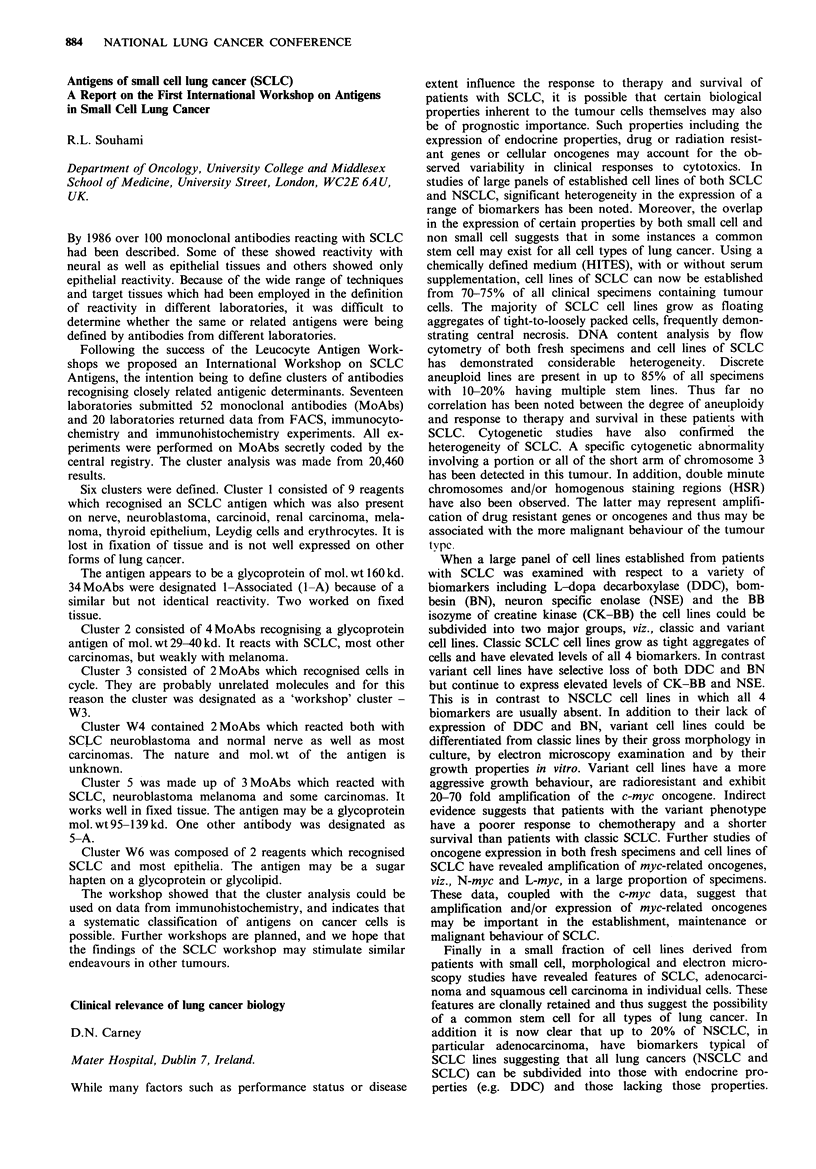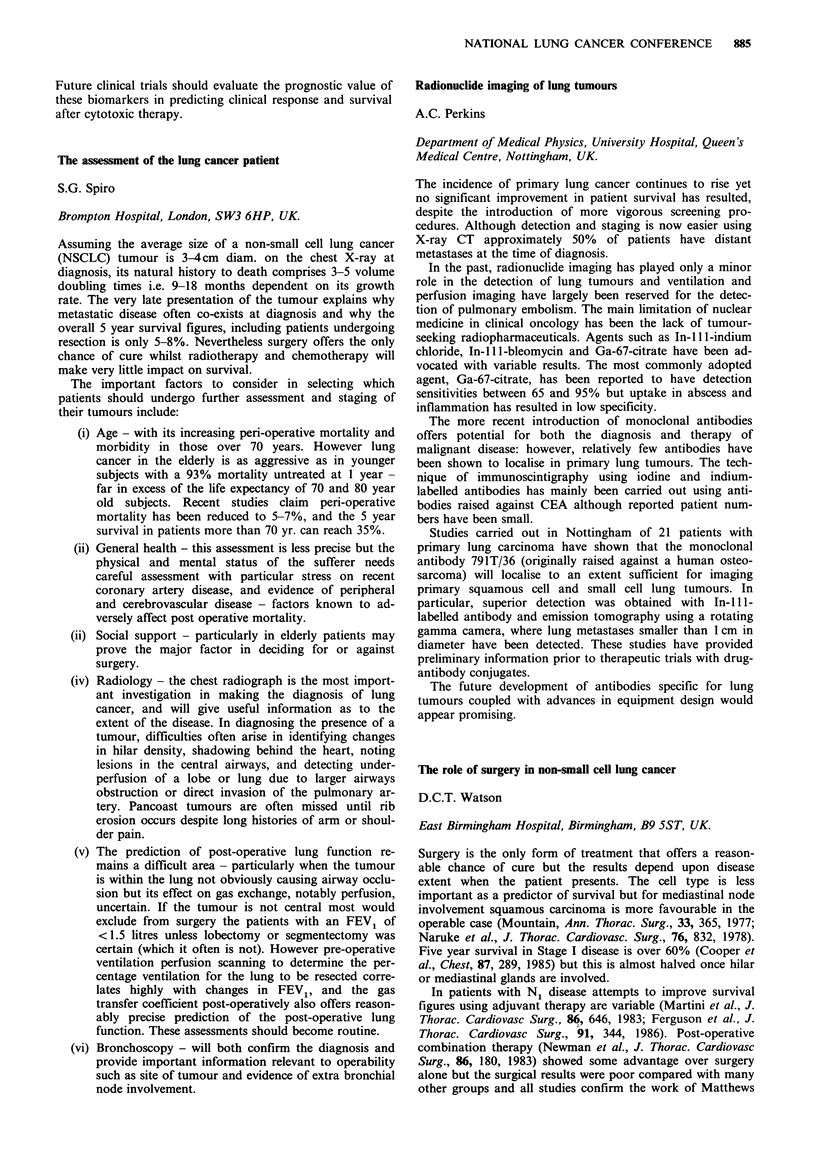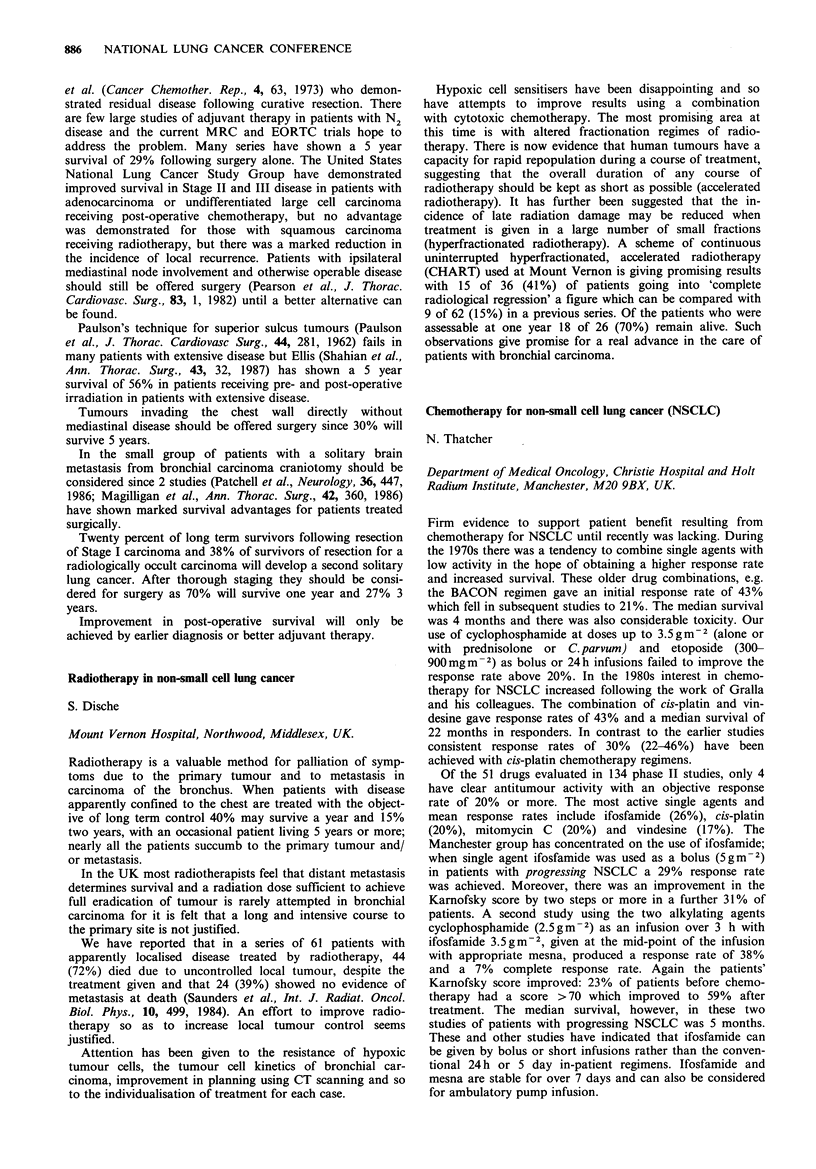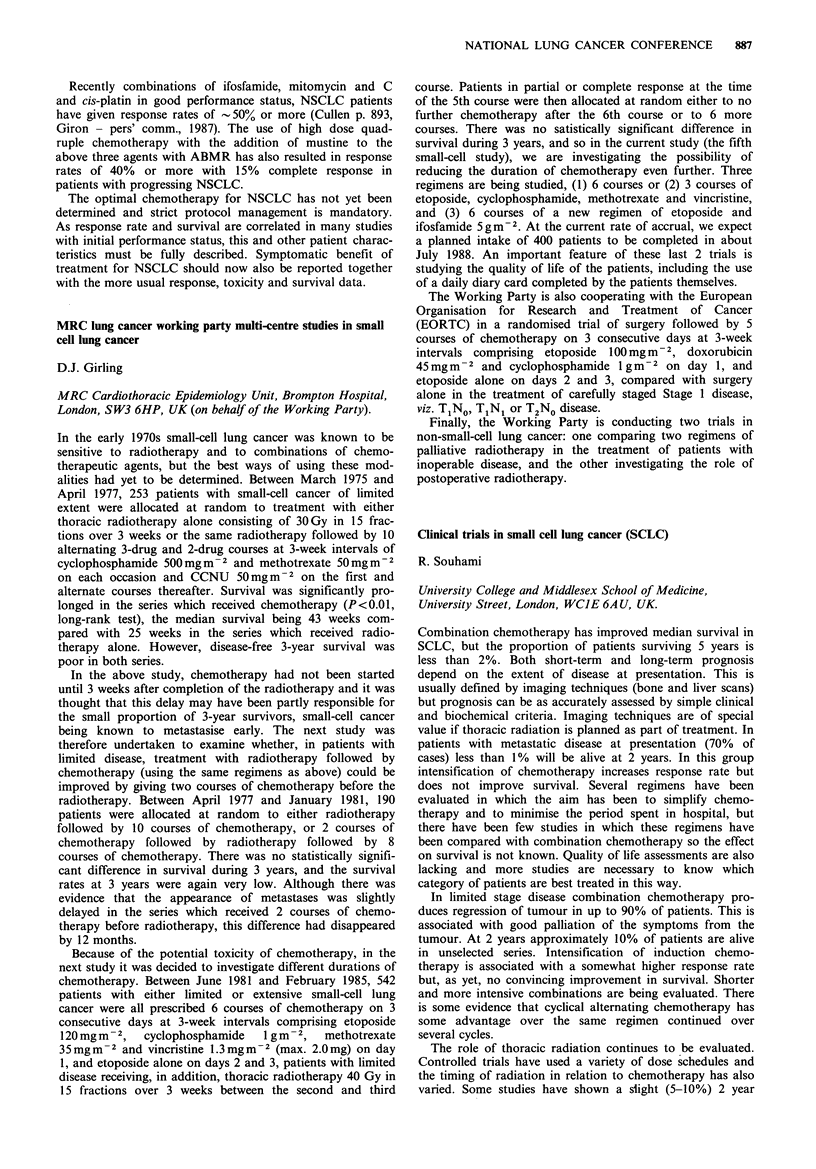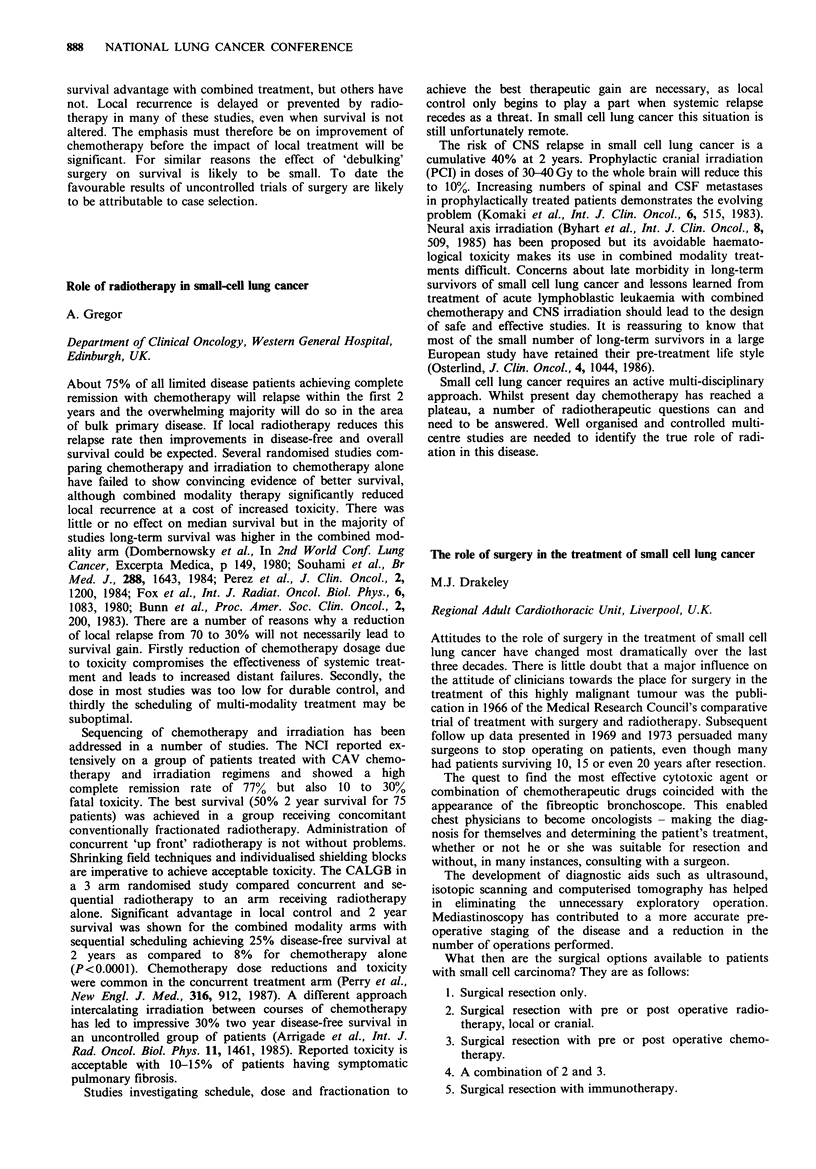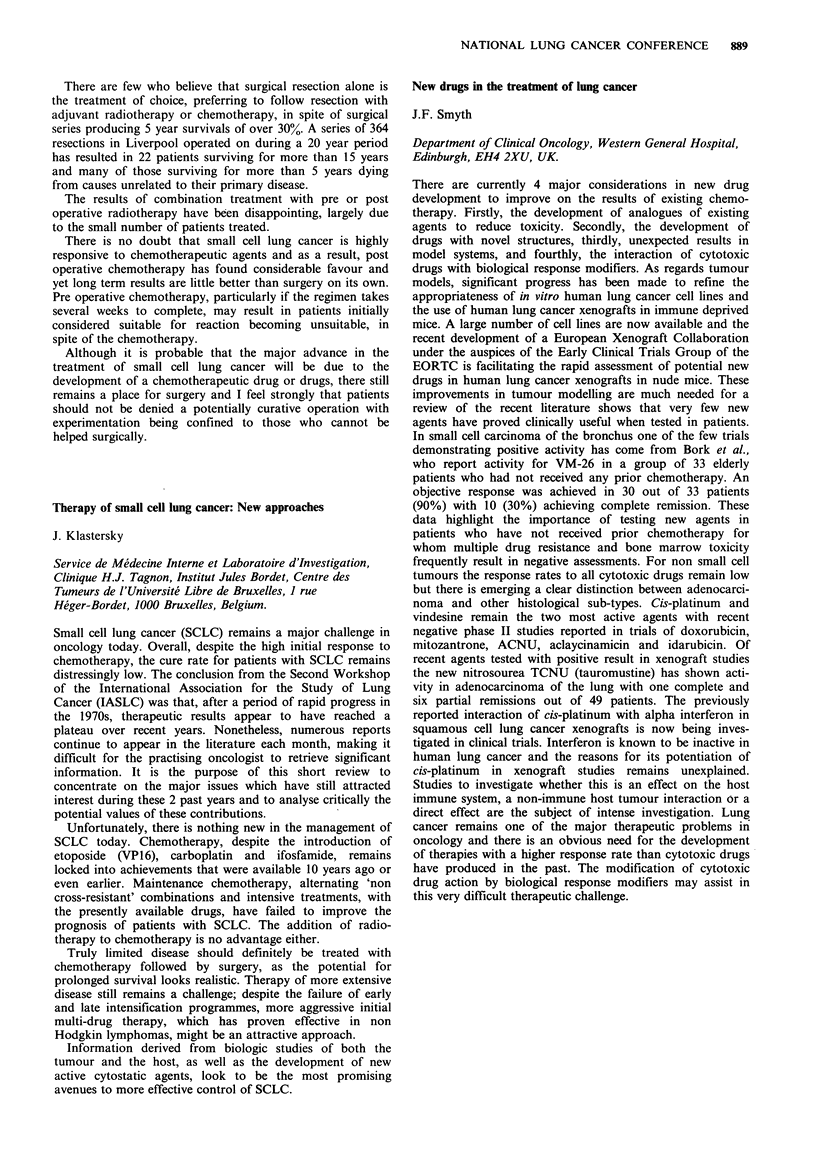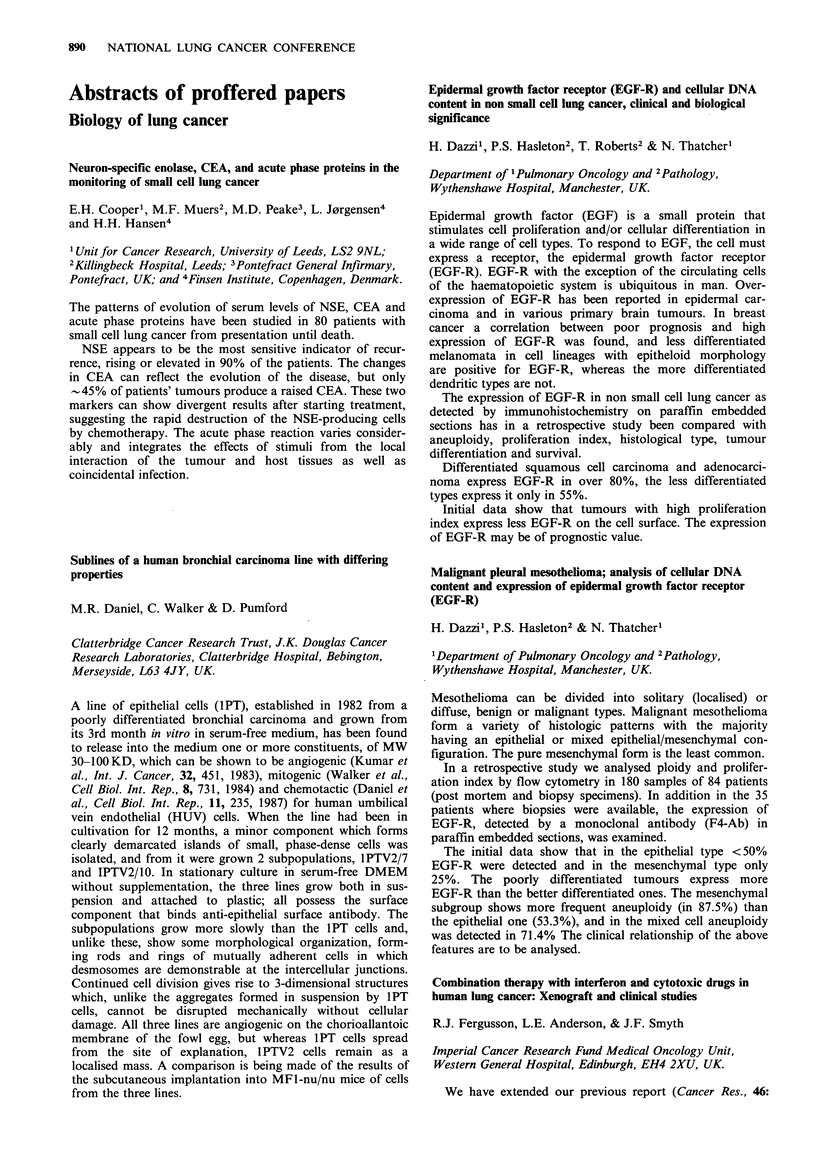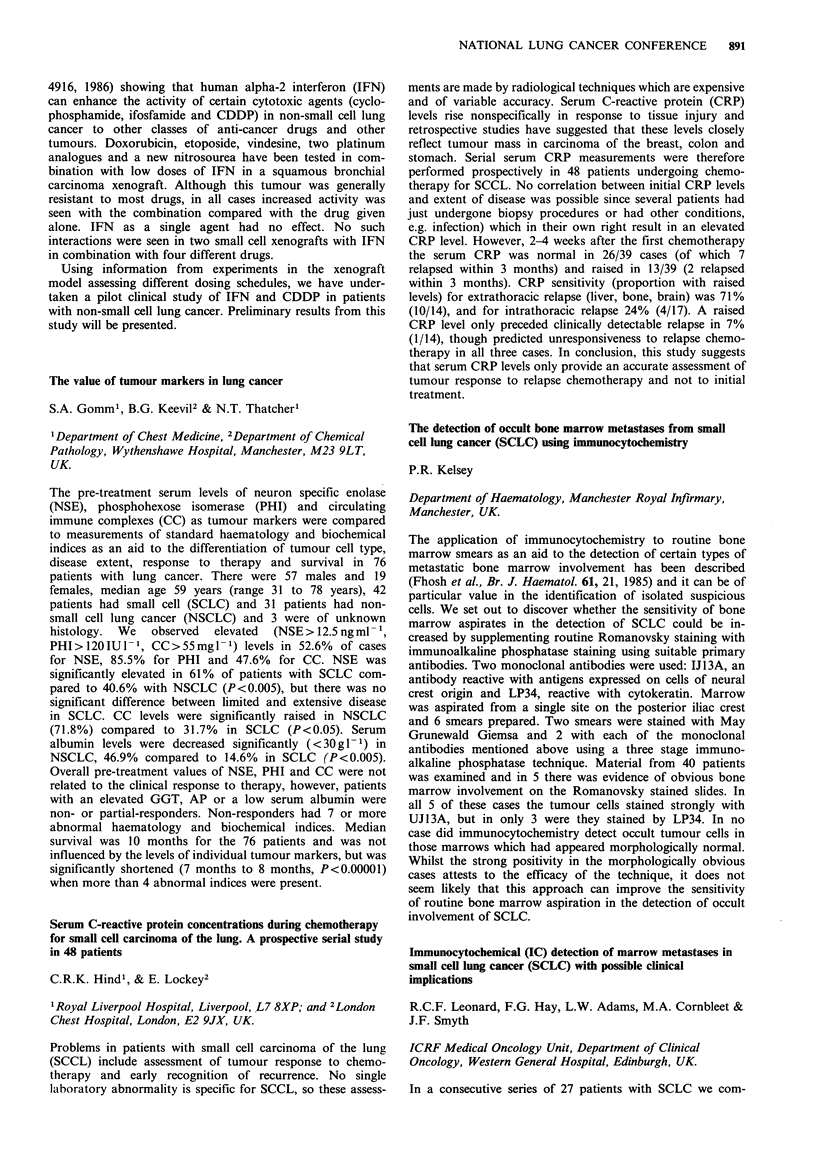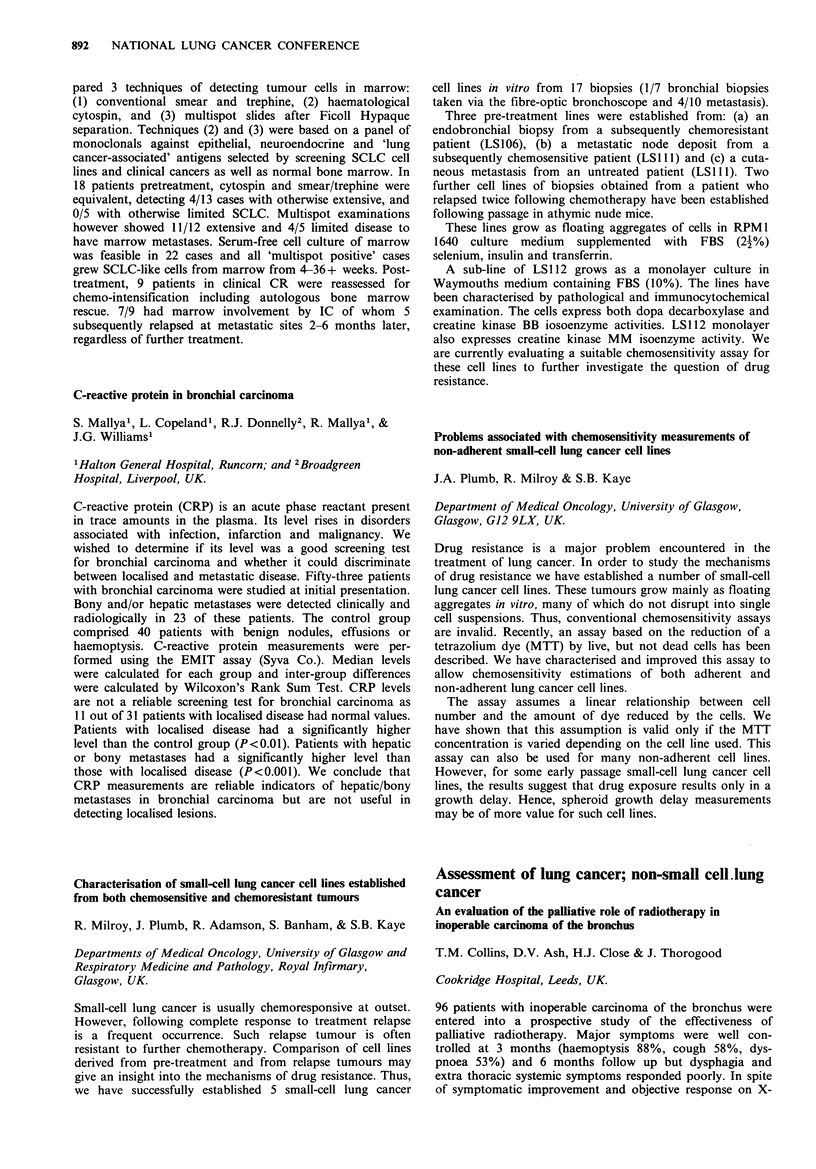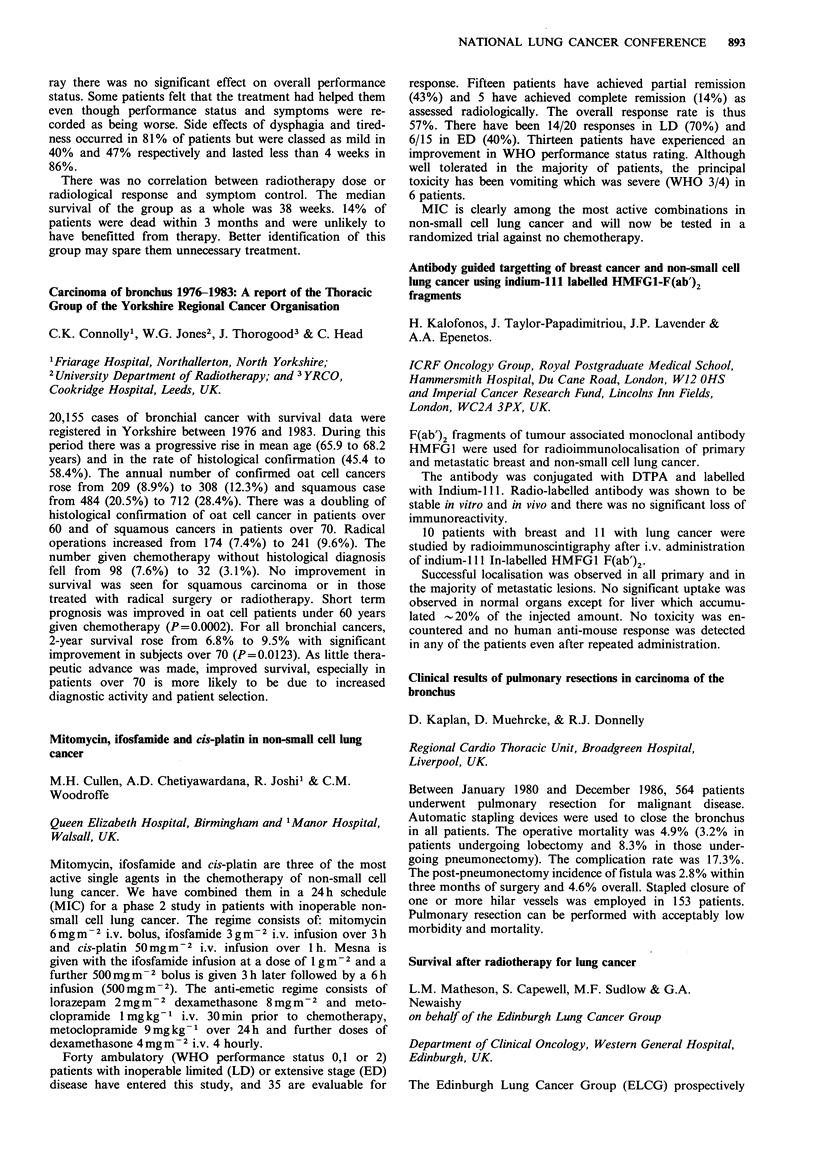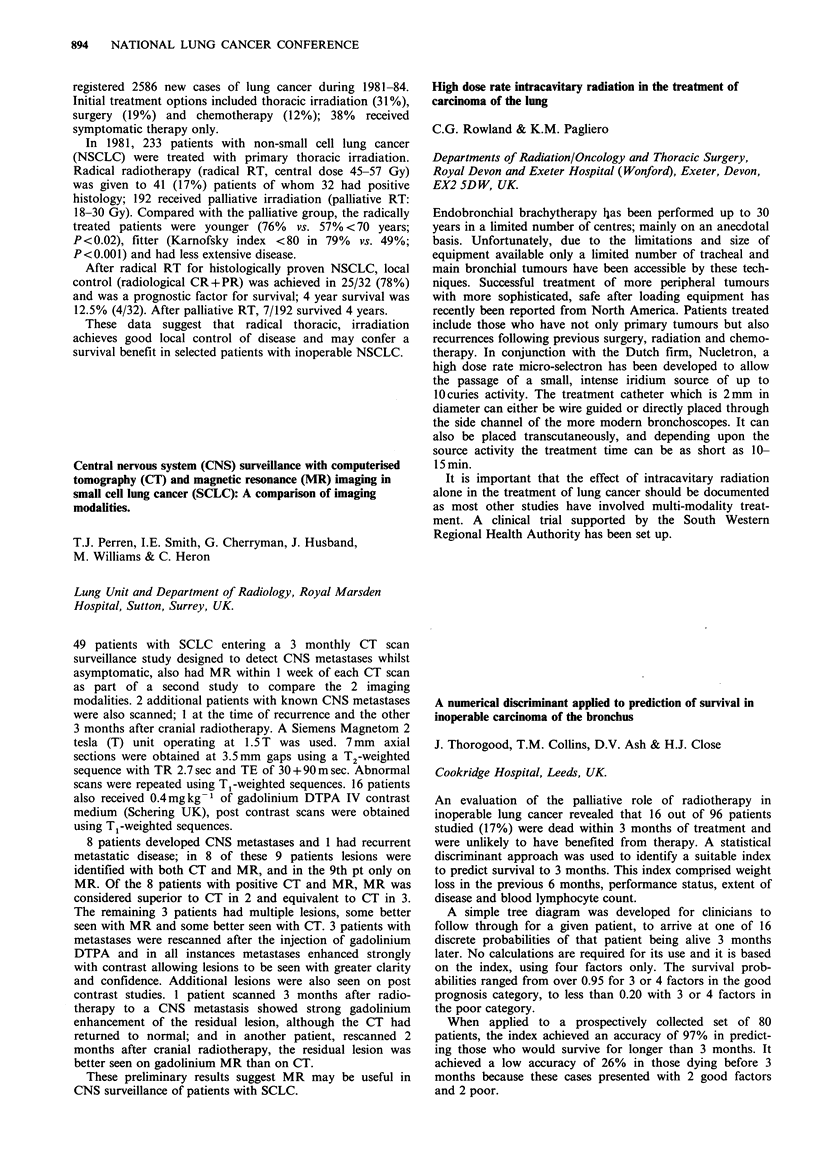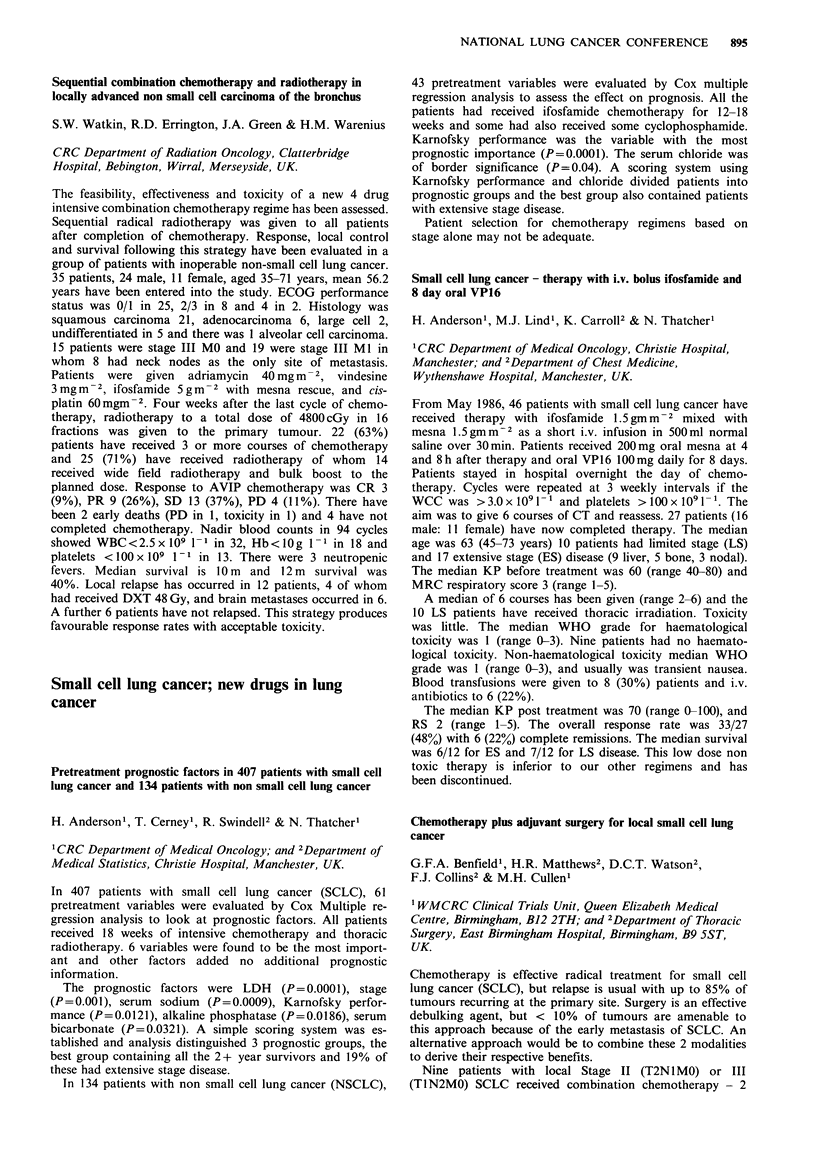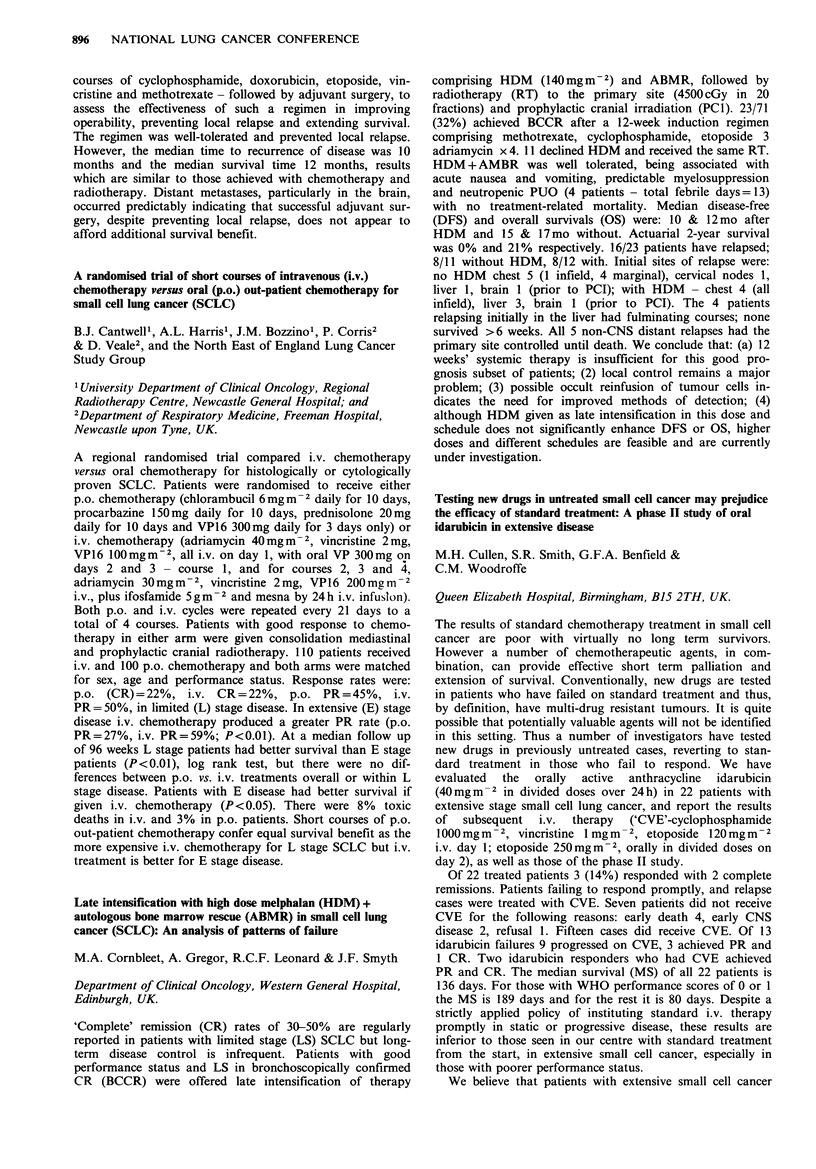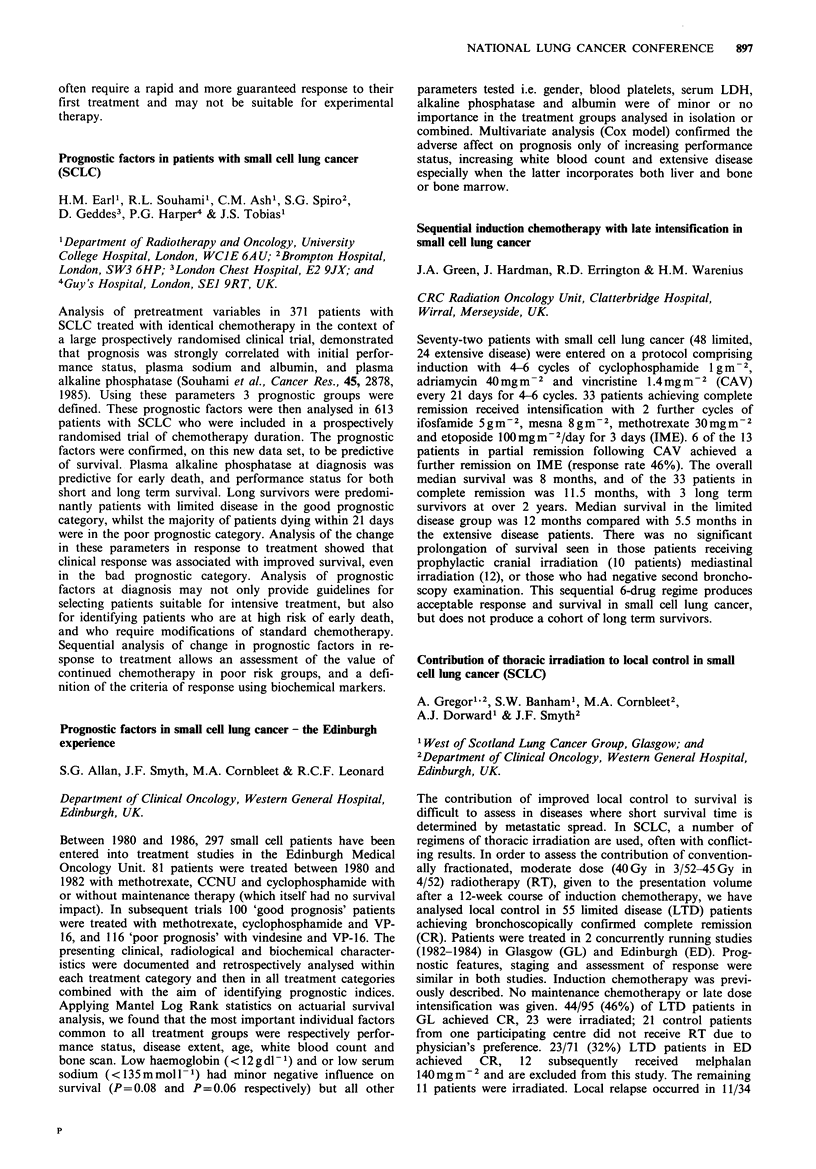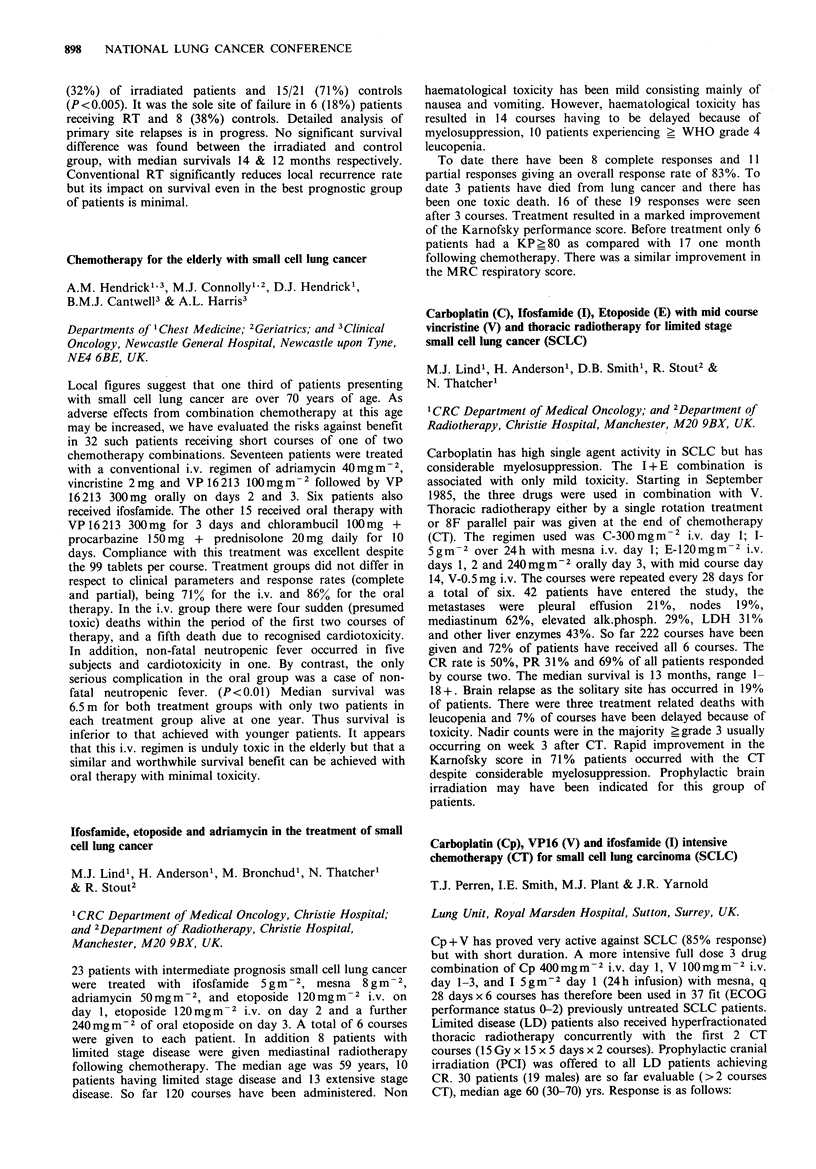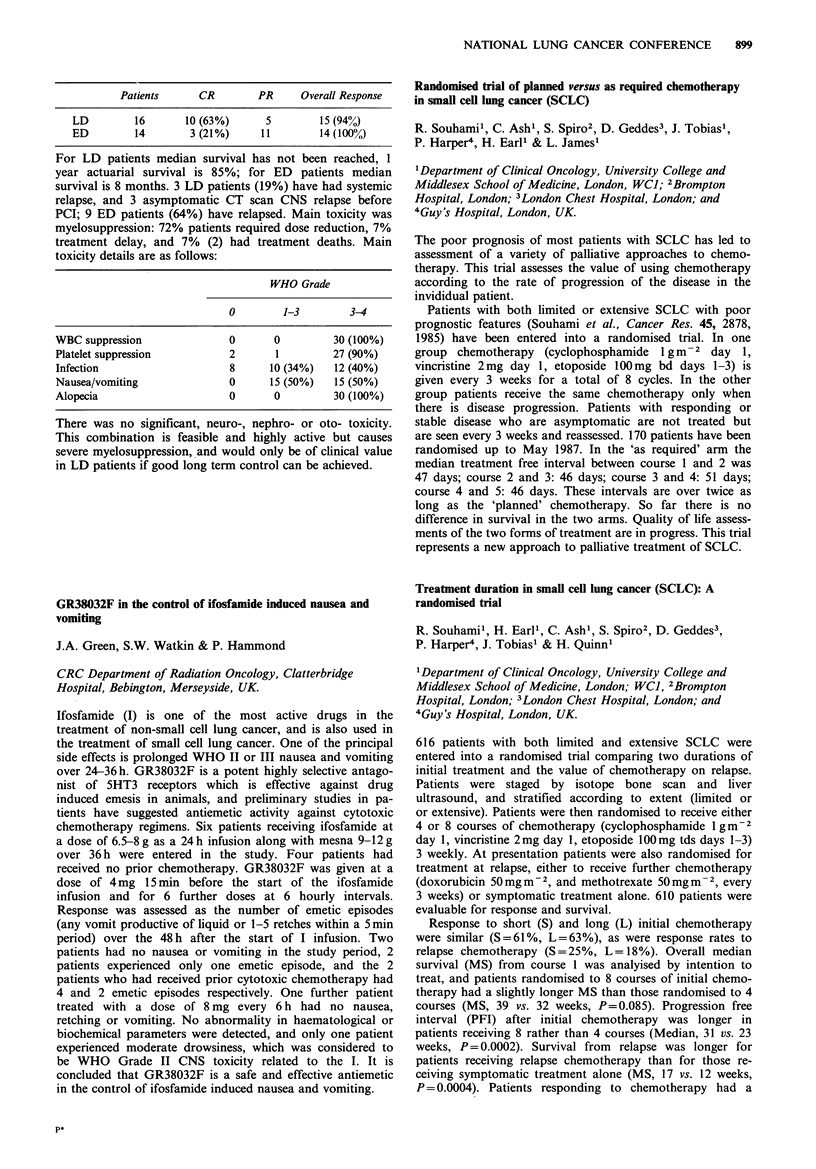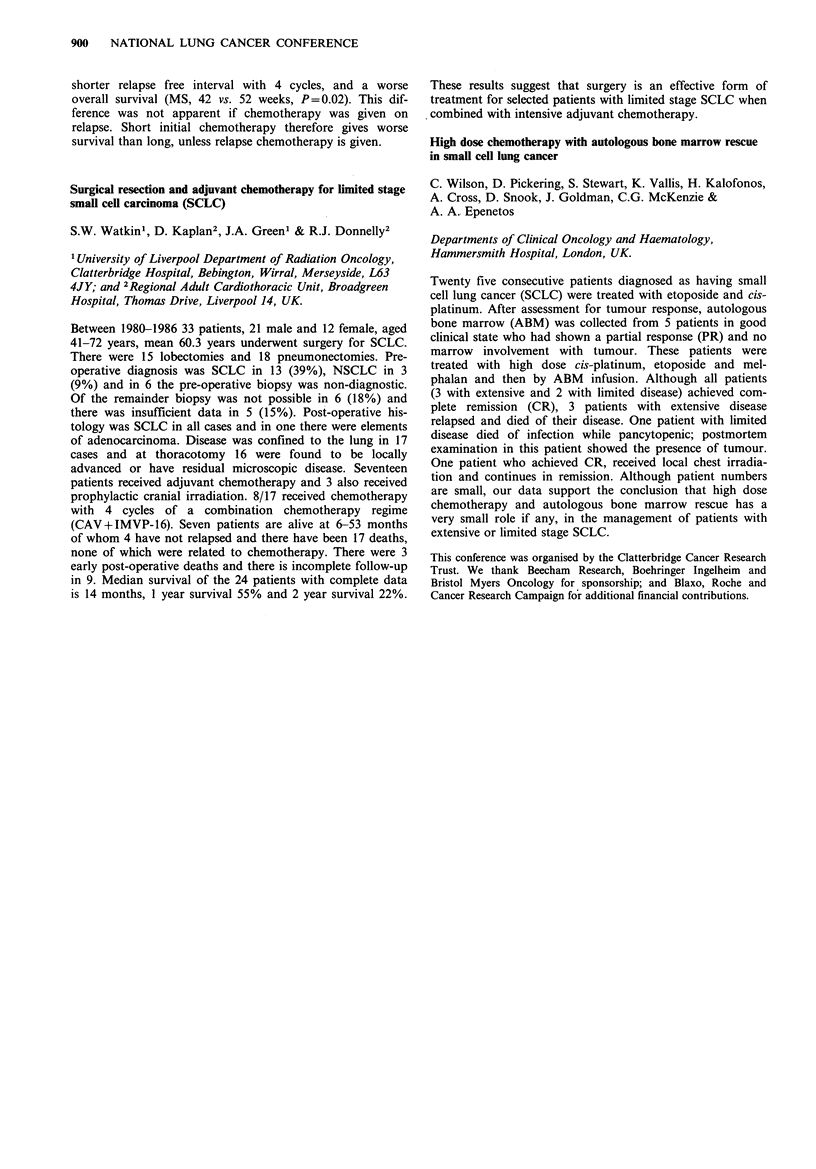# National Lung Conference (September 23-25, 1987). Merseyside, UK. Abstracts.

**Published:** 1987-12

**Authors:** 


Br. J. Cancer (1987), 56, 881–900 © The Macmillan Press, Ltd., 1987

### National Lung Cancer Conference

### (September 23–25, 1987)

*Held at Clatterbridge Hospital, Merseyside, UK.*

### Abstracts of Invited Papers

### The epidemiology of lung cancer in the West of Scotland – Pointers for the future?

C.R. Gillis & D.J. Hole

*West of Scotland Cancer Surveillance Unit, Ruchill Hospital, Glasgow, UK.*

The West of Scotland has some of the highest rates of both lung cancer and heart disease recorded. If an epidemiological study of cigarette smoking and lung cancer showed differences from those previously reported it might point to new approaches. A case-control study of 656 males with twice that number of non-smoking related controls has shown a linear increase in the relative risk of heart disease, but little increase in relative risk of lung cancer above an average exposure of 15–24 cigarettes per day (the cell type determined in 90% of cases studied). Despite the reduction in tar content since levels were first measured, medium tar cigarettes still conferred high relative risk if more than 15 cigarettes per day were smoked. None of these findings are explained by smoking practices peculiar to the West of Scotland. Smokers returned to the risk of lung cancer in non-smokers following 20 years of ex-smoking.

A cohort of 15,399 apparently healthy males and females aged 45–64 was examined between 1972 and 1976 by multiphasic screening for cardiovascular risk including measurement of blood pressure, ECG, serum cholesterol and FEV1. These were entered into the cancer registry in 1972. By December, 1986, 2711 of these have died and the cohort mortality for lung cancer shows, for equal amounts smoked, that the West of Scotland population is at more than twice the absolute risk of lung cancer than any of the cohort studies in the literature.

Also an increasing dose response relationship for lung cancer was found comparing controls, passive smokers (relative risk = 2.4), those who smoked and smokers who lived in households where at least one other member of the household smoked.

The rates of lung cancer in those now living in green field sites in the new towns around Glasgow but who lived within the City of Glasgow in the 1950s were only slightly lower than in Glasgow itself. So the additional risk of lung cancer from either passive smoking or atmospheric pollution does not account for the West of Scotland excess which continues to be observed compared to other Scottish cities.

Serum cholesterol readings from those apparently healthy individuals measured at least 4 years prior to the diagnosis of lung cancer or any other cancer show that high serum cholesterol levels were associated with low rates of lung cancer, contrary to many published findings.

This and the surprising nature of the dose-response relationship allow speculation about the existence of a genetic component in the aetiology.

An epidemiological study has been carried out of knowledge and opinion in Glasgow, Manchester and Leeds about symptoms and signs that may mean cancer. While knowledge improved with increased inputs of education, education itself appeared less important than other variables (age, sex, family experience of cancer, social class) in determining knowledge.

Conference Chairman: J.A. Green, CRC Department of Radiation Oncology, Clatterbridge Hospital, Bebington, Merseyside, L63 4JY, UK (to whom requests for reprints should be sent).
Conference Secretary: S.W. Watkin.

### Education on lung cancer prevention in schools

A. Charlton

*Cancer Research Campaign Education and Child Studies Research Group, University of Manchester, UK.*

Probably the least effective way to prevent lung cancer is to teach about lung cancer prevention. When the link between lung cancer and smoking became clear, it was generally assumed that if people were familiarised with this association they would not smoke.

In a study by the author in 1979, children who smoked were significantly more likely than nonsmokers, to state that smoking can cause lung cancer. Bewley and her colleagues showed that although children could name diseases associated with smoking they did not understand the meaning. Diseases which might strike at the age of 40 or over are much too distant to have any meaning for children. Some children enjoy the added excitement of risk. Others are made very anxious if their parents smoke and lectures about health risks can be very boring.

It is nevertheless imperative that something should be done to prevent children from smoking, as Doll and Peto have shown that the risk of lung cancer increases the earlier smoking is started.

Regular two-yearly surveys carried out by the OPCS now show the prevalence of smoking among children. Nearly 30% of young people are regular smokers at school-leaving age. The prevalence is higher among the less academically inclined. A large CRC-funded study showed that among 16 to 18-year-olds at vocational colleges the prevalence of regular smoking was 31% whilst in the same age group in the sixth forms it was 12%. The same survey showed that children whose parents smoke are nearly twice as likely to be smokers; whilst parents' approval is associated with a seven times increased rate. Only 2% of children would smoke if they thought their best friend was not in agreement.

Young smokers are likely to attend more discos more often than the nonsmokers; to attend gigs more often; to drink alcohol more often. Young smokers are already more likely than nonsmokers to express positive views about smoking e.g. that it calms the nerves, keeps the weight down, gives confidence. Perhaps this is related to advertisement-awareness because smokers were significantly more likely than nonsmokers to have a favourite cigarette advertisement.

The peak age for trying a first cigarette is 9 or 10 years. At 12 or 13 regular smoking becomes established and at 15 or 16 the desire to stop smoking increases.

Smoking control education needs to take place at the critical times; it needs to take into account the children at high risk of becoming smokers and focus on overcoming lifestyle problems.

Above all it must seem relevant to them. A recent CRC study showed that teenage smokers were significantly less likely than nonsmokers to be interested in sport. Health education should take into account where those who are smokers *want* to be; in the discos, in the rock concerts, in the pubs. Health must be compatible with their own lifestyle and background.

### Classification of lung cancer

M.S. Dunnill and K.C. Gatter

*Histopathology Department, John Radcliffe Hospital, Oxford, OX3 9DU, UK.*

For the past twenty years pathologists have classified lung cancer according to the system devised by WHO Kreybeg *et al.* (eds) *Histological typing of lung tumours* (WHO, 1967; *Amer. J. Clin. Pathol.* **77,** 123, 1982). This relied entirely on light microscopical appearances and was detailed, complex and took little account of histogenesis. Recent investigations (Dunnill & Gatter, *Histopathology,* **10,** 461, 1986) using electron microscopy and modern immunocytochemistry have called into question the validity of this classification and have favoured the concept, originally put forward by Willis (In: *Pathology of tumours,* 3rd edn., Butterworths, London, 1960, p. 368), that lung cancer is a single entity, albeit with one cell type often dominating the histological appearance in any given tumour.

To date a total of 161 lung tumours removed at lobectomy or pneumonectomy have been investigated. Portions of tumour tissue taken immediately after removal were (i) snap frozen in liquid nitrogen for immunocytochemistry, (ii) fixed in buffered glutaraldehyde for electron microscopy and (iii) placed in buffered formol saline for processing for light microscopy. Frozen sections were examined, using a three stage immunoperoxidase procedure or the APAAP immunoalkaline phosphatase technique, with monoclonal antibodies against cytokeratins of varying molecular weight, neurally related antigens, carcinoembryonic antigen, vimentin and desmin. Material submitted for electron microscopy was scrutinised for the presence of dense core granules, tonofilaments, mucous secretory vesicles or for an acinar arrangement to the tumour cells. Paraffin sections were stained for mucus using the alcian blue/PAS method and for silver positive granules employing the Grimelius method.

Tumours were classified by light microscopy into one of four categories, *viz.* squamous carcinoma, small cell carcinoma, carcinoids or adenocarcinoma. Immunocytochemical investigation of these tumours showed that those with a clear differentiation towards one of the categories mentioned above tended to have a characteristic immunocytochemical profile. Yet there was considerable cross reactivity. Thus tumours classed as squamous carcinomas often exhibited focal areas which reacted with the antineural antibody UJ13A which typically stains small cell carcinomas. Similarly small cell carcinomas often contained elements which stained positively for high molecular weight keratins. Another finding of interest was that many tumours showed inappropriate co-expression of intermediate filament proteins. Thus some carcinomas reacted with antivimentin or antidesmin antibodies, a feature that is normally found only in connective tissue or muscle. This cellular heterogeneity was further confirmed by examination of ultrastructure and by mucous histochemistry. Many tumours which appeared to be of squamous origin on light microscopy were found to contain dense core granules or mucus-secreting elements.

It is thus apparent that many, perhaps the majority, of lung tumours do not differentiate along one pure cell line. This implies that rigid allocation of lung cancers into WHO categories gives a misleading idea with respect to histogenesis of these tumours. Further support for this comes firstly from biochemical investigation of lung cancers which has shown that many non-small cell cancers contain markers that were once thought to be found only in small cell tumours. Secondly, measurements of cell and nuclear size in so-called small cell tumours has revealed that the term small cell is itself spurious as there is a continuous distribution of cell size from small to large cell tumours.

Thirdly, there is strong epidemiological evidence linking small cell carcinoma with other forms of lung cancer in that all are related to smoking and to uranium exposure.

These findings are most in keeping with the concept that the carcinogenic stimulus affects a pleuripotential basal or reserve cell which then proliferates along one or more pathways. This view would support that of Yesner and Carter (*Clin. Chest Med.* **3,** 257, 1982) who stated that 'all lung cancers are part of a spectrum of differentiation.'

### Historical review

C. Ogilvie

*Royal Liverpool Hospital, Prescot Street, Liverpool, UK.*

On the occasion of the British Lung Cancer Conference, it seemed appropriate to view the history of lung cancer through the eyes of readers of British medical journals. Three journals were selected: the *Quarterly Journal of Medicine* (a journal for general physicians), *Thorax* (a journal for chest physicians and surgeons) and the *British Journal of Cancer* (a journal for oncologists). During the first 80 years of the life of the *Quarterly Journal of Medicine* (1907–1986) and the first 30 years in the life of the other two journals (1947–1977), a total of 222 papers on lung cancer were published. Only 9 of these appeared in the *Quarterly Journal of Medicine* and less than 5% in the other two journals dealt with this commonest of all cancers. In the *British Journal of Cancer,* there were twice as many papers on colon cancer and three times as many on breast cancer. This relative disinterest in lung cancer may be due in part to the fact that, unlike other common cancers, cancer of the lung is in most cases self-inflicted and preventable, but incurable even when early diagnosis is achieved.

Dividing the papers into 5 broad categories, there were 95 on aetiology, 34 on epidemiology, 30 on histocytology, 33 on clinical aspects and 18 on treatment.

The majority of the papers on aetiology related to the analysis of cigarette and urban smoke for carcinogens and the study in small laboratory animals of lung tumours induced by smoke constituents.

Epidemiological studies described the possible relation of lung cancer to smoking, occupation, sex or domicile (urban or rural) but smoking habits were not always taken into account when the effects of other variables were examined. Two thirds of papers in this category were accounts of lung cancer patterns in overseas countries.

Of the 30 papers under the heading of histocytology, 20 dealt with differences in the clinical features, smoking habits, prognosis or treatment of patients with different cell types of lung cancer. Sputum cytology was the subject for most of the remainder.

More than half the 33 clinical papers described metastatic spread of the disease, 10 being accounts of techniques for the recognition of mediastinal involvement. There was only one paper on early diagnosis and none on any aspect of bronchoscopy.

There were 12 papers in *Thorax* on the surgical treatment of lung cancer but only six of the 222 papers in the three journals dealt with medical management. There were no papers on either the prevention or palliation of the disease.

It is concluded that an occasional audit of papers published in medical journals may draw attention to areas of neglect, bearing in mind that research endeavour is not necessarily geared to clinical need. It would seem that in the three journals examined during the period under review, lung cancer was itself an area of comparative neglect, especially with regard to early diagnosis, prevention and palliation.

### Model systems for lung cancer

P.R. Twentyman

*MRC Clinical Oncology and Radiotherapeutics Unit, Hills Road, Cambridge, UK.*

There is clearly no one model system which can contain all the elements which make up human lung cancer. The appropriate model for any particular study will need to include the essential determinants which provide the biological basis of the phenomenon under investigation. In addition to the neoplastic cell population, a varying degree of differentiation, and a stromal component (cellular and extracellular) will define a tumour mass. The reaction of the host to the presence of the tumour will also, in many cases, influence the behaviour of the tumour cells.

Animals lung tumours have made only a small contribution to date with regard to our understanding of the biology of human lung cancer. Tumours produced in mice and rats by inhalation of carcinogens (including tobacco smoke) are not generally of the same histological types as the common human tumours. More recently, however, animal models bearing a closer histological relationship to the common types of lung cancer have been developed. Much of our current data have been obtained by the use of cell lines or xenograft tumours grown from human clinical material. There is no doubt that production of cell lines or the establismment xenografts entail a high degree of selective pressure and that the final product represents the progeny of only a small proportion of the cells present in the original sample. Under appropriate conditions, however, both cell lines and xenografts retain the chemo- and radiosensitivity characteristics of the original tumour type. Cell lines also maintain many of the cytological, ultra-structural and neuro-endocrine properties of the original material. One must be aware of the possibility, however, that phenomena such as oncogene amplification may reflect events occurring *in vitro* during establishment of the cell line and not in the tumour *in situ*.

In addition to growth as monolayers or floating aggregates of cells, cell lines may also be grown as multicellular tumour spheroids. These three-dimensional balls of cells (up to 1 mm in diameter) may be useful for studies of intercellular communication, factors determining the onset of cell death, and cytotoxic drug penetration.

The problem of acquired drug resistance in small cell lung cancer has prompted several groups to derive drug-resistant cell lines. In the absence of definitive evidence as to the nature of drug resistance as seen in the clinic, it is difficult to assess the relevance of such models. However, some of the protein changes seen in the cell lines do correspond to changes which are believed to have clinical significance in other tumour types. These model systems may help in the development of strategies for dealing with drug-resistant disease, such as identification of drug analogues with greater retention of activity in resistant disease and additional chemicals which can act as 'resistance modifiers'.

### Markers in lung cancer

J.G. Reeve

*MRC Clinical Oncology and Radiotherapeutics Unit, Hills Road, Cambridge, UK.*

The development of sensitive radioimmunoassays for enzymes and peptide hormones, the production of monoclonal antibodies to cell surface determinants and investigations into the molecular genetics of lung cancer have generated a wealth of data regarding the expression of endocrine markers, membrane antigens, cytogenetic markers and the amplification/expression of oncogenes in lung tumour cell lines and patient material. It is now clear from such studies that a considerable overlap exists between SCLC and NSCLC particularly with respect to the expression of endocrine-related markers and cell surface protein phenotype, suggesting perhaps a common cellular origin for all types of lung cancer. Importantly, the expression of endocrine properties by some but not all SCLC and NSCLC tumours has prompted clinical trials in which, in addition to routine histology, lung tumours are classified according to their endocrine status to determine whether there is a correlation between the expression of endocrine properties and response to therapy and survival.

Whilst the presence or absence of a given biochemical marker may be of dubious value in the differential diagnosis of lung cancer, certain biomarkers, e.g. neuron specific enolase and calcitonin, may be useful in monitoring therapy, particularly in patients with SCLC and in the diagnosis of CNS metastases. However, despite a voluminous literature, a well defined role for markers in decisions on therapy has not yet been presented. There is, for example, no single marker for which elevated levels can be detected in all patients; some markers, e.g. bombesin, cannot be detected in serum or plasma; the absolute level of a marker does not indicate tumour burden in the individual patient; marker levels do not necessarily increase upon relapse and if they do rise, this does not always indicate relapse. Furthermore, few studies indicate that measurements of markers might improve on the prognostic evaluation as normally performed with staging and performance status.

The search for reliable tumour markers has prompted many groups to develop monoclonal antibodies (MoAbs) to membrane determinants. Although many MoAbs have been generated, their widespread use has been limited by the lack of specificity of individual antibodies and by the problem of antigenic heterogeneity. However, some may be of value in the differential diagnosis of lung cancer, in monitoring therapy, in the detection of metastases and in the *in vitro* elimination of tumour cells from contaminated marrow prior to autologous reinfusion.

While considerable heterogeneity exists within a given tumour with respect to many biochemical markers, deletion of chromosome 3p has been detected with remarkable consistency in SCLC using specific DNA probes. Further studies are required in order to confirm whether this deletion also occurs at high frequency in NSCLC tumours and whether a recessive tumour suppressor gene may be involved in the development of lung cancer in general.

The identification of selected biomarkers, e.g. bombesin, as autocrine growth factors has led to speculation that interacting neuroendocrine signals are involved in SCLC growth control. A monoclonal antibody to bombesin has been produced which inhibits the growth of SCLC both *in vitro* and in xenografts, and is now entering a phase I clinical trial, providing one example of how disruption of signal transduction may represent a new form of endocrine therapy for lung cancer.

**Antigens of small cell lung cancer (SCLC)**
**A Report on the First International Workshop on Antigens in Small Cell Lung Cancer**

R.L. Souhami

*Department of Oncology, University College and Middlesex School of Medicine, University Street, London, WC2E 6AU, UK.*

By 1986 over 100 monoclonal antibodies reacting with SCLC had been described. Some of these showed reactivity with neural as well as epithelial tissues and others showed only epithelial reactivity. Because of the wide range of techniques and target tissues which had been employed in the definition of reactivity in different laboratories, it was difficult to determine whether the same or related antigens were being defined by antibodies from different laboratories.

Following the success of the Leucocyte Antigen Workshops we proposed an International Workshop on SCLC Antigens, the intention being to define clusters of antibodies recognising closely related antigenic determinants. Seventeen laboratories submitted 52 monoclonal antibodies (MoAbs) and 20 laboratories returned data from FACS, immunocytochemistry and immunohistochemistry experiments. All experiments were performed on MoAbs secretly coded by the central registry. The cluster analysis was made from 20,460 results.

Six clusters were defined. Cluster 1 consisted of 9 reagents which recognised an SCLC antigen which was also present on nerve, neuroblastoma, carcinoid, renal carcinoma, melanoma, thyroid epithelium, Leydig cells and erythrocytes. It is lost in fixation of tissue and is not well expressed on other forms of lung cancer.

The antigen appears to be a glycoprotein of mol. wt 160 kd. 34 MoAbs were designated 1-Associated (1-A) because of a similar but not identical reactivity. Two worked on fixed tissue.

Cluster 2 consisted of 4 MoAbs recognising a glycoprotein antigen of mol. wt 29–40 kd. It reacts with SCLC, most other carcinomas, but weakly with melanoma.

Cluster 3 consisted of 2 MoAbs which recognised cells in cycle. They are probably unrelated molecules and for this reason the cluster was designated as a 'workshop' cluster – W3.

Cluster W4 contained 2 MoAbs which reacted both with SCLC neuroblastoma and normal nerve as well as most carcinomas. The nature and mol. wt of the antigen is unknown.

Cluster 5 was made up of 3 MoAbs which reacted with SCLC, neuroblastoma melanoma and some carcinomas. It works well in fixed tissue. The antigen may be a glycoprotein mol. wt 95–139 kd. One other antibody was designated as 5-A.

Cluster W6 was composed of 2 reagents which recognised SCLC and most epithelia. The antigen may be a sugar hapten on a glycoprotein or glycolipid.

The workshop showed that the cluster analysis could be used on data from immunohistochemistry, and indicates that a systematic classification of antigens on cancer cells is possible. Further workshops are planned, and we hope that the findings of the SCLC workshop may stimulate similar endeavours in other tumours.

**Clinical relevance of lung cancer biology**

D.N. Carney

*Mater Hospital, Dublin 7, Ireland.*

While many factors such as performance status or disease extent influence the response to therapy and survival of patients with SCLC, it is possible that certain biological properties inherent to the tumour cells themselves may also be of prognostic importance. Such properties including the expression of endocrine properties, drug or radiation resistant genes or cellular oncogenes may account for the observed variability in clinical responses to cytotoxics. In studies of large panels of established cell lines of both SCLC and NSCLC, significant heterogeneity in the expression of a range of biomarkers has been noted. Moreover, the overlap in the expression of certain properties by both small cell and non small cell suggests that in some instances a common stem cell may exist for all cell types of lung cancer. Using a chemically defined medium (HITES), with or without serum supplementation, cell lines of SCLC can now be established from 70–75% of all clinical specimens containing tumour cells. The majority of SCLC cell lines grow as floating aggregates of tight-to-loosely packed cells, frequently demonstrating central necrosis. DNA content analysis by flow cytometry of both fresh specimens and cell lines of SCLC has demonstrated considerable heterogeneity. Discrete aneuploid lines are present in up to 85% of all specimens with 10–20% having multiple stem lines. Thus far no correlation has been noted between the degree of aneuploidy and response to therapy and survival in these patients with SCLC. Cytogenetic studies have also confirmed the heterogeneity of SCLC. A specific cytogenetic abnormality involving a portion or all of the short arm of chromosome 3 has been detected in this tumour. In addition, double minute chromosomes and/or homogenous staining regions (HSR) have also been observed. The latter may represent amplification of drug resistant genes or oncogenes and thus may be associated with the more malignant behaviour of the tumour type.

When a large panel of cell lines established from patients with SCLC was examined with respect to a variety of biomarkers including L-dopa decarboxylase (DDC), bombesin (BN), neuron specific enolase (NSE) and the BB isozyme of creatine kinase (CK-BB) the cell lines could be subdivided into two major groups, *viz.*, classic and variant cell lines. Classic SCLC cell lines grow as tight aggregates of cells and have elevated levels of all 4 biomarkers. In contrast variant cell lines have selective loss of both DDC and BN but continue to express elevated levels of CK-BB and NSE. This is in contrast to NSCLC cell lines in which all 4 biomarkers are usually absent. In addition to their lack of expression of DDC and BN, variant cell lines could be differentiated from classic lines by their gross morphology in culture, by electron microscopy examination and by their growth properties *in vitro*. Variant cell lines have a more aggressive growth behaviour, are radioresistant and exhibit 20–70 fold amplification of the *c-myc* oncogene. Indirect evidence suggests that patients with the variant phenotype have a poorer response to chemotherapy and a shorter survival than patients with classic SCLC. Further studies of oncogene expression in both fresh specimens and cell lines of SCLC have revealed amplification of *myc*-related oncogenes, *viz.*, N-*myc* and L-*myc*, in a large proportion of specimens. These data, coupled with the c-*myc* data, suggest that amplification and/or expression of *myc*-related oncogenes may be important in the establishment, maintenance or malignant behaviour of SCLC.

Finally in a small fraction of cell lines derived from patients with small cell, morphological and electron microscopy studies have revealed features of SCLC, adenocarcinoma and squamous cell carcinoma in individual cells. These features are clonally retained and thus suggest the possibility of a common stem cell for all types of lung cancer. In addition it is now clear that up to 20% of NSCLC, in particular adenocarcinoma, have biomarkers typical of SCLC lines suggesting that all lung cancers (NSCLC and SCLC) can be subdivided into those with endocrine properties (e.g. DDC) and those lacking those properties.

Future clinical trials should evaluate the prognostic value of these biomarkers in predicting clinical response and survival after cytotoxic therapy.

### The assessment of the lung cancer patient

S.G. Spiro

*Brompton Hospital, London, SW3 6HP, UK.*

Assuming the average size of a non-small cell lung cancer (NSCLC) tumour is 3–4 cm diam. on the chest X-ray at diagnosis, its natural history to death comprises 3–5 volume doubling times i.e. 9–18 months dependent on its growth rate. The very late presentation of the tumour explains why metastatic disease often co-exists at diagnosis and why the overall 5 year survival figures, including patients undergoing resection is only 5–8%. Nevertheless surgery offers the only chance of cure whilst radiotherapy and chemotherapy will make very little impact on survival.

The important factors to consider in selecting which patients should undergo further assessment and staging of their tumours include:

(i) Age – with its increasing peri-operative mortality and morbidity in those over 70 years. However lung cancer in the elderly is as aggressive as in younger subjects with a 93% mortality untreated at 1 year – far in excess of the life expectancy of 70 and 80 year old subjects. Recent studies claim peri-operative mortality has been reduced to 5–7%, and the 5 year survival in patients more than 70 yr. can reach 35%.

(ii) General health – this assessment is less precise but the physical and mental status of the sufferer needs careful assessment with particular stress on recent coronary artery disease, and evidence of peripheral and cerebrovascular disease – factors known to adversely affect post operative mortality.

(ii) Social support – particularly in elderly patients may prove the major factor in deciding for or against surgery.

(iv) Radiology – the chest radiograph is the most important investigation in making the diagnosis of lung cancer, and will give useful information as to the extent of the disease. In diagnosing the presence of a tumour, difficulties often arise in identifying changes in hilar density, shadowing behind the heart, noting lesions in the central airways, and detecting underperfusion of a lobe or lung due to larger airways obstruction or direct invasion of the pulmonary artery. Pancoast tumours are often missed until rib erosion occurs despite long histories of arm or shoulder pain.

(v) The prediction of post-operative lung function remains a difficult area – particularly when the tumour is within the lung not obviously causing airway occlusion but its effect on gas exchange, notably perfusion, uncertain. If the tumour is not central most would exclude from surgery the patients with an $FEV_1$ of $<1.5$ litres unless lobectomy or segmentectomy was certain (which it often is not). However pre-operative ventilation perfusion scanning to determine the percentage ventilation for the lung to be resected correlates highly with changes in $FEV_1$, and the gas transfer coefficient post-operatively also offers reasonably precise prediction of the post-operative lung function. These assessments should become routine.

(vi) Bronchoscopy – will both confirm the diagnosis and provide important information relevant to operability such as site of tumour and evidence of extra bronchial node involvement.

### Radionuclide imaging of lung tumours

A.C. Perkins

*Department of Medical Physics, University Hospital, Queen's Medical Centre, Nottingham, UK.*

The incidence of primary lung cancer continues to rise yet no significant improvement in patient survival has resulted, despite the introduction of more vigorous screening procedures. Although detection and staging is now easier using X-ray CT approximately 50% of patients have distant metastases at the time of diagnosis.

In the past, radionuclide imaging has played only a minor role in the detection of lung tumours and ventilation and perfusion imaging have largely been reserved for the detection of pulmonary embolism. The main limitation of nuclear medicine in clinical oncology has been the lack of tumour-seeking radiopharmaceuticals. Agents such as In-111-indium chloride, In-111-bleomycin and Ga-67-citrate have been advocated with variable results. The most commonly adopted agent, Ga-67-citrate, has been reported to have detection sensitivities between 65 and 95% but uptake in abscess and inflammation has resulted in low specificity.

The more recent introduction of monoclonal antibodies offers potential for both the diagnosis and therapy of malignant disease: however, relatively few antibodies have been shown to localise in primary lung tumours. The technique of immunoscintigraphy using iodine and indium-labelled antibodies has mainly been carried out using antibodies raised against CEA although reported patient numbers have been small.

Studies carried out in Nottingham of 21 patients with primary lung carcinoma have shown that the monoclonal antibody 791T/36 (originally raised against a human osteosarcoma) will localise to an extent sufficient for imaging primary squamous cell and small cell lung tumours. In particular, superior detection was obtained with In-111-labelled antibody and emission tomography using a rotating gamma camera, where lung metastases smaller than 1 cm in diameter have been detected. These studies have provided preliminary information prior to therapeutic trials with drug-antibody conjugates.

The future development of antibodies specific for lung tumours coupled with advances in equipment design would appear promising.

### The role of surgery in non-small cell lung cancer

D.C.T. Watson

*East Birmingham Hospital, Birmingham, B9 5ST, UK.*

Surgery is the only form of treatment that offers a reasonable chance of cure but the results depend upon disease extent when the patient presents. The cell type is less important as a predictor of survival but for mediastinal node involvement squamous carcinoma is more favourable in the operable case (Mountain, *Ann. Thorac. Surg.*, **33**, 365, 1977; Naruke *et al.*, *J. Thorac. Cardiovasc. Surg.*, **76**, 832, 1978). Five year survival in Stage I disease is over 60% (Cooper *et al.*, *Chest*, **87**, 289, 1985) but this is almost halved once hilar or mediastinal glands are involved.

In patients with $N_1$ disease attempts to improve survival figures using adjuvant therapy are variable (Martini *et al.*, *J. Thorac. Cardiovasc Surg.*, **86**, 646, 1983; Ferguson *et al.*, *J. Thorac. Cardiovasc Surg.*, **91**, 344, 1986). Post-operative combination therapy (Newman *et al.*, *J. Thorac. Cardiovasc Surg.*, **86**, 180, 1983) showed some advantage over surgery alone but the surgical results were poor compared with many other groups and all studies confirm the work of Matthews

*et al.* (*Cancer Chemother. Rep.*, **4**, 63, 1973) who demonstrated residual disease following curative resection. There are few large studies of adjuvant therapy in patients with $N_2$ disease and the current MRC and EORTC trials hope to address the problem. Many series have shown a 5 year survival of 29% following surgery alone. The United States National Lung Cancer Study Group have demonstrated improved survival in Stage II and III disease in patients with adenocarcinoma or undifferentiated large cell carcinoma receiving post-operative chemotherapy, but no advantage was demonstrated for those with squamous carcinoma receiving radiotherapy, but there was a marked reduction in the incidence of local recurrence. Patients with ipsilateral mediastinal node involvement and otherwise operable disease should still be offered surgery (Pearson *et al.*, *J. Thorac. Cardiovasc. Surg.*, **83**, 1, 1982) until a better alternative can be found.

Paulson's technique for superior sulcus tumours (Paulson *et al.*, *J. Thorac. Cardiovasc Surg.*, **44**, 281, 1962) fails in many patients with extensive disease but Ellis (Shahian *et al.*, *Ann. Thorac. Surg.*, **43**, 32, 1987) has shown a 5 year survival of 56% in patients receiving pre- and post-operative irradiation in patients with extensive disease.

Tumours invading the chest wall directly without mediastinal disease should be offered surgery since 30% will survive 5 years.

In the small group of patients with a solitary brain metastasis from bronchial carcinoma craniotomy should be considered since 2 studies (Patchell *et al.*, *Neurology*, **36**, 447, 1986; Magilligan *et al.*, *Ann. Thorac. Surg.*, **42**, 360, 1986) have shown marked survival advantages for patients treated surgically.

Twenty percent of long term survivors following resection of Stage I carcinoma and 38% of survivors of resection for a radiologically occult carcinoma will develop a second solitary lung cancer. After thorough staging they should be considered for surgery as 70% will survive one year and 27% 3 years.

Improvement in post-operative survival will only be achieved by earlier diagnosis or better adjuvant therapy.

**Radiotherapy in non-small cell lung cancer**

S. Dische

*Mount Vernon Hospital, Northwood, Middlesex, UK.*

Radiotherapy is a valuable method for palliation of symptoms due to the primary tumour and to metastasis in carcinoma of the bronchus. When patients with disease apparently confined to the chest are treated with the objective of long term control 40% may survive a year and 15% two years, with an occasional patient living 5 years or more; nearly all the patients succumb to the primary tumour and/or metastasis.

In the UK most radiotherapists feel that distant metastasis determines survival and a radiation dose sufficient to achieve full eradication of tumour is rarely attempted in bronchial carcinoma for it is felt that a long and intensive course to the primary site is not justified.

We have reported that in a series of 61 patients with apparently localised disease treated by radiotherapy, 44 (72%) died due to uncontrolled local tumour, despite the treatment given and that 24 (39%) showed no evidence of metastasis at death (Saunders *et al.*, *Int. J. Radiat. Oncol. Biol. Phys.*, **10**, 499, 1984). An effort to improve radiotherapy so as to increase local tumour control seems justified.

Attention has been given to the resistance of hypoxic tumour cells, the tumour cell kinetics of bronchial carcinoma, improvement in planning using CT scanning and so to the individualisation of treatment for each case.

Hypoxic cell sensitisers have been disappointing and so have attempts to improve results using a combination with cytotoxic chemotherapy. The most promising area at this time is with altered fractionation regimes of radiotherapy. There is now evidence that human tumours have a capacity for rapid repopulation during a course of treatment, suggesting that the overall duration of any course of radiotherapy should be kept as short as possible (accelerated radiotherapy). It has further been suggested that the incidence of late radiation damage may be reduced when treatment is given in a large number of small fractions (hyperfractionated radiotherapy). A scheme of continuous uninterrupted hyperfractionated, accelerated radiotherapy (CHART) used at Mount Vernon is giving promising results with 15 of 36 (41%) of patients going into 'complete radiological regression' a figure which can be compared with 9 of 62 (15%) in a previous series. Of the patients who were assessable at one year 18 of 26 (70%) remain alive. Such observations give promise for a real advance in the care of patients with bronchial carcinoma.

**Chemotherapy for non-small cell lung cancer (NSCLC)**

N. Thatcher

*Department of Medical Oncology, Christie Hospital and Holt Radium Institute, Manchester, M20 9BX, UK.*

Firm evidence to support patient benefit resulting from chemotherapy for NSCLC until recently was lacking. During the 1970s there was a tendency to combine single agents with low activity in the hope of obtaining a higher response rate and increased survival. These older drug combinations, e.g. the BACON regimen gave an initial response rate of 43% which fell in subsequent studies to 21%. The median survival was 4 months and there was also considerable toxicity. Our use of cyclophosphamide at doses up to $3.5\,g\,m^{-2}$ (alone or with prednisolone or *C.parvum)* and etoposide (300–900 $mg\,m^{-2}$) as bolus or 24 h infusions failed to improve the response rate above 20%. In the 1980s interest in chemotherapy for NSCLC increased following the work of Gralla and his colleagues. The combination of *cis*-platin and vindesine gave response rates of 43% and a median survival of 22 months in responders. In contrast to the earlier studies consistent response rates of 30% (22–46%) have been achieved with *cis*-platin chemotherapy regimens.

Of the 51 drugs evaluated in 134 phase II studies, only 4 have clear antitumour activity with an objective response rate of 20% or more. The most active single agents and mean response rates include ifosfamide (26%), *cis*-platin (20%), mitomycin C (20%) and vindesine (17%). The Manchester group has concentrated on the use of ifosfamide; when single agent ifosfamide was used as a bolus ($5\,g\,m^{-2}$) in patients with *progressing* NSCLC a 29% response rate was achieved. Moreover, there was an improvement in the Karnofsky score by two steps or more in a further 31% of patients. A second study using the two alkylating agents cyclophosphamide ($2.5\,g\,m^{-2}$) as an infusion over 3 h with ifosfamide $3.5\,g\,m^{-2}$, given at the mid-point of the infusion with appropriate mesna, produced a response rate of 38% and a 7% complete response rate. Again the patients' Karnofsky score improved: 23% of patients before chemotherapy had a score $>70$ which improved to 59% after treatment. The median survival, however, in these two studies of patients with progressing NSCLC was 5 months. These and other studies have indicated that ifosfamide can be given by bolus or short infusions rather than the conventional 24 h or 5 day in-patient regimens. Ifosfamide and mesna are stable for over 7 days and can also be considered for ambulatory pump infusion.

Recently combinations of ifosfamide, mitomycin and C and *cis*-platin in good performance status, NSCLC patients have given response rates of ~50% or more (Cullen p. 893, Giron – pers' comm., 1987). The use of high dose quadruple chemotherapy with the addition of mustine to the above three agents with ABMR has also resulted in response rates of 40% or more with 15% complete response in patients with progressing NSCLC.

The optimal chemotherapy for NSCLC has not yet been determined and strict protocol management is mandatory. As response rate and survival are correlated in many studies with initial performance status, this and other patient characteristics must be fully described. Symptomatic benefit of treatment for NSCLC should now also be reported together with the more usual response, toxicity and survival data.

### MRC lung cancer working party multi-centre studies in small cell lung cancer

D.J. Girling

*MRC Cardiothoracic Epidemiology Unit, Brompton Hospital, London, SW3 6HP, UK (on behalf of the Working Party).*

In the early 1970s small-cell lung cancer was known to be sensitive to radiotherapy and to combinations of chemotherapeutic agents, but the best ways of using these modalities had yet to be determined. Between March 1975 and April 1977, 253 patients with small-cell cancer of limited extent were allocated at random to treatment with either thoracic radiotherapy alone consisting of 30 Gy in 15 fractions over 3 weeks or the same radiotherapy followed by 10 alternating 3-drug and 2-drug courses at 3-week intervals of cyclophosphamide 500 mg m$^{-2}$ and methotrexate 50 mg m$^{-2}$ on each occasion and CCNU 50 mg m$^{-2}$ on the first and alternate courses thereafter. Survival was significantly prolonged in the series which received chemotherapy ($P<0.01$, long-rank test), the median survival being 43 weeks compared with 25 weeks in the series which received radiotherapy alone. However, disease-free 3-year survival was poor in both series.

In the above study, chemotherapy had not been started until 3 weeks after completion of the radiotherapy and it was thought that this delay may have been partly responsible for the small proportion of 3-year survivors, small-cell cancer being known to metastasise early. The next study was therefore undertaken to examine whether, in patients with limited disease, treatment with radiotherapy followed by chemotherapy (using the same regimens as above) could be improved by giving two courses of chemotherapy before the radiotherapy. Between April 1977 and January 1981, 190 patients were allocated at random to either radiotherapy followed by 10 courses of chemotherapy, or 2 courses of chemotherapy followed by radiotherapy followed by 8 courses of chemotherapy. There was no statistically significant difference in survival during 3 years, and the survival rates at 3 years were again very low. Although there was evidence that the appearance of metastases was slightly delayed in the series which received 2 courses of chemotherapy before radiotherapy, this difference had disappeared by 12 months.

Because of the potential toxicity of chemotherapy, in the next study it was decided to investigate different durations of chemotherapy. Between June 1981 and February 1985, 542 patients with either limited or extensive small-cell lung cancer were all prescribed 6 courses of chemotherapy on 3 consecutive days at 3-week intervals comprising etoposide 120 mg m$^{-2}$, cyclophosphamide 1 g m$^{-2}$, methotrexate 35 mg m$^{-2}$ and vincristine 1.3 mg m$^{-2}$ (max. 2.0 mg) on day 1, and etoposide alone on days 2 and 3, patients with limited disease receiving, in addition, thoracic radiotherapy 40 Gy in 15 fractions over 3 weeks between the second and third course. Patients in partial or complete response at the time of the 5th course were then allocated at random either to no further chemotherapy after the 6th course or to 6 more courses. There was no satistically significant difference in survival during 3 years, and so in the current study (the fifth small-cell study), we are investigating the possibility of reducing the duration of chemotherapy even further. Three regimens are being studied, (1) 6 courses or (2) 3 courses of etoposide, cyclophosphamide, methotrexate and vincristine, and (3) 6 courses of a new regimen of etoposide and ifosfamide 5 g m$^{-2}$. At the current rate of accrual, we expect a planned intake of 400 patients to be completed in about July 1988. An important feature of these last 2 trials is studying the quality of life of the patients, including the use of a daily diary card completed by the patients themselves.

The Working Party is also cooperating with the European Organisation for Research and Treatment of Cancer (EORTC) in a randomised trial of surgery followed by 5 courses of chemotherapy on 3 consecutive days at 3-week intervals comprising etoposide 100 mg m$^{-2}$, doxorubicin 45 mg m$^{-2}$ and cyclophosphamide 1 g m$^{-2}$ on day 1, and etoposide alone on days 2 and 3, compared with surgery alone in the treatment of carefully staged Stage 1 disease, *viz.* $T_1N_0$, $T_1N_1$ or $T_2N_0$ disease.

Finally, the Working Party is conducting two trials in non-small-cell lung cancer: one comparing two regimens of palliative radiotherapy in the treatment of patients with inoperable disease, and the other investigating the role of postoperative radiotherapy.

### Clinical trials in small cell lung cancer (SCLC)

R. Souhami

*University College and Middlesex School of Medicine, University Street, London, WC1E 6AU, UK.*

Combination chemotherapy has improved median survival in SCLC, but the proportion of patients surviving 5 years is less than 2%. Both short-term and long-term prognosis depend on the extent of disease at presentation. This is usually defined by imaging techniques (bone and liver scans) but prognosis can be as accurately assessed by simple clinical and biochemical criteria. Imaging techniques are of special value if thoracic radiation is planned as part of treatment. In patients with metastatic disease at presentation (70% of cases) less than 1% will be alive at 2 years. In this group intensification of chemotherapy increases response rate but does not improve survival. Several regimens have been evaluated in which the aim has been to simplify chemotherapy and to minimise the period spent in hospital, but there have been few studies in which these regimens have been compared with combination chemotherapy so the effect on survival is not known. Quality of life assessments are also lacking and more studies are necessary to know which category of patients are best treated in this way.

In limited stage disease combination chemotherapy produces regression of tumour in up to 90% of patients. This is associated with good palliation of the symptoms from the tumour. At 2 years approximately 10% of patients are alive in unselected series. Intensification of induction chemotherapy is associated with a somewhat higher response rate but, as yet, no convincing improvement in survival. Shorter and more intensive combinations are being evaluated. There is some evidence that cyclical alternating chemotherapy has some advantage over the same regimen continued over several cycles.

The role of thoracic radiation continues to be evaluated. Controlled trials have used a variety of dose schedules and the timing of radiation in relation to chemotherapy has also varied. Some studies have shown a slight (5–10%) 2 year survival advantage with combined treatment, but others have not. Local recurrence is delayed or prevented by radiotherapy in many of these studies, even when survival is not altered. The emphasis must therefore be on improvement of chemotherapy before the impact of local treatment will be significant. For similar reasons the effect of 'debulking' surgery on survival is likely to be small. To date the favourable results of uncontrolled trials of surgery are likely to be attributable to case selection.

### Role of radiotherapy in small-cell lung cancer

A. Gregor

*Department of Clinical Oncology, Western General Hospital, Edinburgh, UK.*

About 75% of all limited disease patients achieving complete remission with chemotherapy will relapse within the first 2 years and the overwhelming majority will do so in the area of bulk primary disease. If local radiotherapy reduces this relapse rate then improvements in disease-free and overall survival could be expected. Several randomised studies comparing chemotherapy and irradiation to chemotherapy alone have failed to show convincing evidence of better survival, although combined modality therapy significantly reduced local recurrence at a cost of increased toxicity. There was little or no effect on median survival but in the majority of studies long-term survival was higher in the combined modality arm (Dombernowsky *et al.*, In *2nd World Conf. Lung Cancer*, Excerpta Medica, p 149, 1980; Souhami *et al.*, *Br Med. J.*, **288**, 1643, 1984; Perez *et al.*, *J. Clin. Oncol.*, **2**, 1200, 1984; Fox *et al.*, *Int. J. Radiat. Oncol. Biol. Phys.*, **6**, 1083, 1980; Bunn *et al.*, *Proc. Amer. Soc. Clin. Oncol.*, **2**, 200, 1983). There are a number of reasons why a reduction of local relapse from 70 to 30% will not necessarily lead to survival gain. Firstly reduction of chemotherapy dosage due to toxicity compromises the effectiveness of systemic treatment and leads to increased distant failures. Secondly, the dose in most studies was too low for durable control, and thirdly the scheduling of multi-modality treatment may be suboptimal.

Sequencing of chemotherapy and irradiation has been addressed in a number of studies. The NCI reported extensively on a group of patients treated with CAV chemotherapy and irradiation regimens and showed a high complete remission rate of 77% but also 10 to 30% fatal toxicity. The best survival (50% 2 year survival for 75 patients) was achieved in a group receiving concomitant conventionally fractionated radiotherapy. Administration of concurrent 'up front' radiotherapy is not without problems. Shrinking field techniques and individualised shielding blocks are imperative to achieve acceptable toxicity. The CALGB in a 3 arm randomised study compared concurrent and sequential radiotherapy to an arm receiving radiotherapy alone. Significant advantage in local control and 2 year survival was shown for the combined modality arms with sequential scheduling achieving 25% disease-free survival at 2 years as compared to 8% for chemotherapy alone ($P < 0.0001$). Chemotherapy dose reductions and toxicity were common in the concurrent treatment arm (Perry *et al.*, *New Engl. J. Med.*, **316**, 912, 1987). A different approach intercalating irradiation between courses of chemotherapy has led to impressive 30% two year disease-free survival in an uncontrolled group of patients (Arrigade *et al.*, *Int. J. Rad. Oncol. Biol. Phys.* **11**, 1461, 1985). Reported toxicity is acceptable with 10–15% of patients having symptomatic pulmonary fibrosis.

Studies investigating schedule, dose and fractionation to achieve the best therapeutic gain are necessary, as local control only begins to play a part when systemic relapse recedes as a threat. In small cell lung cancer this situation is still unfortunately remote.

The risk of CNS relapse in small cell lung cancer is a cumulative 40% at 2 years. Prophylactic cranial irradiation (PCI) in doses of 30–40 Gy to the whole brain will reduce this to 10%. Increasing numbers of spinal and CSF metastases in prophylactically treated patients demonstrates the evolving problem (Komaki *et al.*, *Int. J. Clin. Oncol.*, **6**, 515, 1983). Neural axis irradiation (Byhart *et al.*, *Int. J. Clin. Oncol.*, **8**, 509, 1985) has been proposed but its avoidable haematological toxicity makes its use in combined modality treatments difficult. Concerns about late morbidity in long-term survivors of small cell lung cancer and lessons learned from treatment of acute lymphoblastic leukaemia with combined chemotherapy and CNS irradiation should lead to the design of safe and effective studies. It is reassuring to know that most of the small number of long-term survivors in a large European study have retained their pre-treatment life style (Osterlind, *J. Clin. Oncol.*, **4**, 1044, 1986).

Small cell lung cancer requires an active multi-disciplinary approach. Whilst present day chemotherapy has reached a plateau, a number of radiotherapeutic questions can and need to be answered. Well organised and controlled multicentre studies are needed to identify the true role of radiation in this disease.

### The role of surgery in the treatment of small cell lung cancer

M.J. Drakeley

*Regional Adult Cardiothoracic Unit, Liverpool, U.K.*

Attitudes to the role of surgery in the treatment of small cell lung cancer have changed most dramatically over the last three decades. There is little doubt that a major influence on the attitude of clinicians towards the place for surgery in the treatment of this highly malignant tumour was the publication in 1966 of the Medical Research Council's comparative trial of treatment with surgery and radiotherapy. Subsequent follow up data presented in 1969 and 1973 persuaded many surgeons to stop operating on patients, even though many had patients surviving 10, 15 or even 20 years after resection.

The quest to find the most effective cytotoxic agent or combination of chemotherapeutic drugs coincided with the appearance of the fibreoptic bronchoscope. This enabled chest physicians to become oncologists – making the diagnosis for themselves and determining the patient's treatment, whether or not he or she was suitable for resection and without, in many instances, consulting with a surgeon.

The development of diagnostic aids such as ultrasound, isotopic scanning and computerised tomography has helped in eliminating the unnecessary exploratory operation. Mediastinoscopy has contributed to a more accurate pre-operative staging of the disease and a reduction in the number of operations performed.

What then are the surgical options available to patients with small cell carcinoma? They are as follows:

1. Surgical resection only.
2. Surgical resection with pre or post operative radiotherapy, local or cranial.
3. Surgical resection with pre or post operative chemotherapy.
4. A combination of 2 and 3.
5. Surgical resection with immunotherapy.

There are few who believe that surgical resection alone is the treatment of choice, preferring to follow resection with adjuvant radiotherapy or chemotherapy, in spite of surgical series producing 5 year survivals of over 30%. A series of 364 resections in Liverpool operated on during a 20 year period has resulted in 22 patients surviving for more than 15 years and many of those surviving for more than 5 years dying from causes unrelated to their primary disease.

The results of combination treatment with pre or post operative radiotherapy have been disappointing, largely due to the small number of patients treated.

There is no doubt that small cell lung cancer is highly responsive to chemotherapeutic agents and as a result, post operative chemotherapy has found considerable favour and yet long term results are little better than surgery on its own. Pre operative chemotherapy, particularly if the regimen takes several weeks to complete, may result in patients initially considered suitable for reaction becoming unsuitable, in spite of the chemotherapy.

Although it is probable that the major advance in the treatment of small cell lung cancer will be due to the development of a chemotherapeutic drug or drugs, there still remains a place for surgery and I feel strongly that patients should not be denied a potentially curative operation with experimentation being confined to those who cannot be helped surgically.

### Therapy of small cell lung cancer: New approaches

J. Klastersky

*Service de Médecine Interne et Laboratoire d'Investigation, Clinique H.J. Tagnon, Institut Jules Bordet, Centre des Tumeurs de l'Université Libre de Bruxelles, 1 rue Héger-Bordet, 1000 Bruxelles, Belgium.*

Small cell lung cancer (SCLC) remains a major challenge in oncology today. Overall, despite the high initial response to chemotherapy, the cure rate for patients with SCLC remains distressingly low. The conclusion from the Second Workshop of the International Association for the Study of Lung Cancer (IASLC) was that, after a period of rapid progress in the 1970s, therapeutic results appear to have reached a plateau over recent years. Nonetheless, numerous reports continue to appear in the literature each month, making it difficult for the practising oncologist to retrieve significant information. It is the purpose of this short review to concentrate on the major issues which have still attracted interest during these 2 past years and to analyse critically the potential values of these contributions.

Unfortunately, there is nothing new in the management of SCLC today. Chemotherapy, despite the introduction of etoposide (VP16), carboplatin and ifosfamide, remains locked into achievements that were available 10 years ago or even earlier. Maintenance chemotherapy, alternating 'non cross-resistant' combinations and intensive treatments, with the presently available drugs, have failed to improve the prognosis of patients with SCLC. The addition of radiotherapy to chemotherapy is no advantage either.

Truly limited disease should definitely be treated with chemotherapy followed by surgery, as the potential for prolonged survival looks realistic. Therapy of more extensive disease still remains a challenge; despite the failure of early and late intensification programmes, more aggressive initial multi-drug therapy, which has proven effective in non Hodgkin lymphomas, might be an attractive approach.

Information derived from biologic studies of both the tumour and the host, as well as the development of new active cytostatic agents, look to be the most promising avenues to more effective control of SCLC.

### New drugs in the treatment of lung cancer

J.F. Smyth

*Department of Clinical Oncology, Western General Hospital, Edinburgh, EH4 2XU, UK.*

There are currently 4 major considerations in new drug development to improve on the results of existing chemotherapy. Firstly, the development of analogues of existing agents to reduce toxicity. Secondly, the development of drugs with novel structures, thirdly, unexpected results in model systems, and fourthly, the interaction of cytotoxic drugs with biological response modifiers. As regards tumour models, significant progress has been made to refine the appropriateness of *in vitro* human lung cancer cell lines and the use of human lung cancer xenografts in immune deprived mice. A large number of cell lines are now available and the recent development of a European Xenograft Collaboration under the auspices of the Early Clinical Trials Group of the EORTC is facilitating the rapid assessment of potential new drugs in human lung cancer xenografts in nude mice. These improvements in tumour modelling are much needed for a review of the recent literature shows that very few new agents have proved clinically useful when tested in patients. In small cell carcinoma of the bronchus one of the few trials demonstrating positive activity has come from Bork *et al.*, who report activity for VM-26 in a group of 33 elderly patients who had not received any prior chemotherapy. An objective response was achieved in 30 out of 33 patients (90%) with 10 (30%) achieving complete remission. These data highlight the importance of testing new agents in patients who have not received prior chemotherapy for whom multiple drug resistance and bone marrow toxicity frequently result in negative assessments. For non small cell tumours the response rates to all cytotoxic drugs remain low but there is emerging a clear distinction between adenocarcinoma and other histological sub-types. *Cis*-platinum and vindesine remain the two most active agents with recent negative phase II studies reported in trials of doxorubicin, mitozantrone, ACNU, aclaycinamicin and idarubicin. Of recent agents tested with positive result in xenograft studies the new nitrosourea TCNU (tauromustine) has shown activity in adenocarcinoma of the lung with one complete and six partial remissions out of 49 patients. The previously reported interaction of *cis*-platinum with alpha interferon in squamous cell lung cancer xenografts is now being investigated in clinical trials. Interferon is known to be inactive in human lung cancer and the reasons for its potentiation of *cis*-platinum in xenograft studies remains unexplained. Studies to investigate whether this is an effect on the host immune system, a non-immune host tumour interaction or a direct effect are the subject of intense investigation. Lung cancer remains one of the major therapeutic problems in oncology and there is an obvious need for the development of therapies with a higher response rate than cytotoxic drugs have produced in the past. The modification of cytotoxic drug action by biological response modifiers may assist in this very difficult therapeutic challenge.

### Abstracts of proffered papers

### Biology of lung cancer

### Neuron-specific enolase, CEA, and acute phase proteins in the monitoring of small cell lung cancer

E.H. Cooper[1], M.F. Muers[2], M.D. Peake[3], L. Jørgensen[4] and H.H. Hansen[4]

[1]*Unit for Cancer Research, University of Leeds, LS2 9NL;* [2]*Killingbeck Hospital, Leeds;* [3]*Pontefract General Infirmary, Pontefract, UK; and* [4]*Finsen Institute, Copenhagen, Denmark.*

The patterns of evolution of serum levels of NSE, CEA and acute phase proteins have been studied in 80 patients with small cell lung cancer from presentation until death.

NSE appears to be the most sensitive indicator of recurrence, rising or elevated in 90% of the patients. The changes in CEA can reflect the evolution of the disease, but only ~45% of patients' tumours produce a raised CEA. These two markers can show divergent results after starting treatment, suggesting the rapid destruction of the NSE-producing cells by chemotherapy. The acute phase reaction varies considerably and integrates the effects of stimuli from the local interaction of the tumour and host tissues as well as coincidental infection.

### Sublines of a human bronchial carcinoma line with differing properties

M.R. Daniel, C. Walker & D. Pumford

*Clatterbridge Cancer Research Trust, J.K. Douglas Cancer Research Laboratories, Clatterbridge Hospital, Bebington, Merseyside, L63 4JY, UK.*

A line of epithelial cells (1PT), established in 1982 from a poorly differentiated bronchial carcinoma and grown from its 3rd month *in vitro* in serum-free medium, has been found to release into the medium one or more constituents, of MW 30–100 KD, which can be shown to be angiogenic (Kumar *et al., Int. J. Cancer,* **32,** 451, 1983), mitogenic (Walker *et al., Cell Biol. Int. Rep.,* **8,** 731, 1984) and chemotactic (Daniel *et al., Cell Biol. Int. Rep.,* **11,** 235, 1987) for human umbilical vein endothelial (HUV) cells. When the line had been in cultivation for 12 months, a minor component which forms clearly demarcated islands of small, phase-dense cells was isolated, and from it were grown 2 subpopulations, 1PTV2/7 and IPTV2/10. In stationary culture in serum-free DMEM without supplementation, the three lines grow both in suspension and attached to plastic; all possess the surface component that binds anti-epithelial surface antibody. The subpopulations grow more slowly than the 1PT cells and, unlike these, show some morphological organization, forming rods and rings of mutually adherent cells in which desmosomes are demonstrable at the intercellular junctions. Continued cell division gives rise to 3-dimensional structures which, unlike the aggregates formed in suspension by 1PT cells, cannot be disrupted mechanically without cellular damage. All three lines are angiogenic on the chorioallantoic membrane of the fowl egg, but whereas 1PT cells spread from the site of explanation, 1PTV2 cells remain as a localised mass. A comparison is being made of the results of the subcutaneous implantation into MF1-nu/nu mice of cells from the three lines.

### Epidermal growth factor receptor (EGF-R) and cellular DNA content in non small cell lung cancer, clinical and biological significance

H. Dazzi[1], P.S. Hasleton[2], T. Roberts[2] & N. Thatcher[1]

*Department of* [1]*Pulmonary Oncology and* [2]*Pathology, Wythenshawe Hospital, Manchester, UK.*

Epidermal growth factor (EGF) is a small protein that stimulates cell proliferation and/or cellular differentiation in a wide range of cell types. To respond to EGF, the cell must express a receptor, the epidermal growth factor receptor (EGF-R). EGF-R with the exception of the circulating cells of the haematopoietic system is ubiquitous in man. Overexpression of EGF-R has been reported in epidermal carcinoma and in various primary brain tumours. In breast cancer a correlation between poor prognosis and high expression of EGF-R was found, and less differentiated melanomata in cell lineages with epitheloid morphology are positive for EGF-R, whereas the more differentiated dendritic types are not.

The expression of EGF-R in non small cell lung cancer as detected by immunohistochemistry on paraffin embedded sections has in a retrospective study been compared with aneuploidy, proliferation index, histological type, tumour differentiation and survival.

Differentiated squamous cell carcinoma and adenocarcinoma express EGF-R in over 80%, the less differentiated types express it only in 55%.

Initial data show that tumours with high proliferation index express less EGF-R on the cell surface. The expression of EGF-R may be of prognostic value.

### Malignant pleural mesothelioma; analysis of cellular DNA content and expression of epidermal growth factor receptor (EGF-R)

H. Dazzi[1], P.S. Hasleton[2] & N. Thatcher[1]

[1]*Department of Pulmonary Oncology and* [2]*Pathology, Wythenshawe Hospital, Manchester, UK.*

Mesothelioma can be divided into solitary (localised) or diffuse, benign or malignant types. Malignant mesothelioma form a variety of histologic patterns with the majority having an epithelial or mixed epithelial/mesenchymal configuration. The pure mesenchymal form is the least common.

In a retrospective study we analysed ploidy and proliferation index by flow cytometry in 180 samples of 84 patients (post mortem and biopsy specimens). In addition in the 35 patients where biopsies were available, the expression of EGF-R, detected by a monoclonal antibody (F4-Ab) in paraffin embedded sections, was examined.

The initial data show that in the epithelial type <50% EGF-R were detected and in the mesenchymal type only 25%. The poorly differentiated tumours express more EGF-R than the better differentiated ones. The mesenchymal subgroup shows more frequent aneuploidy (in 87.5%) than the epithelial one (53.3%), and in the mixed cell aneuploidy was detected in 71.4% The clinical relationship of the above features are to be analysed.

### Combination therapy with interferon and cytotoxic drugs in human lung cancer: Xenograft and clinical studies

R.J. Fergusson, L.E. Anderson, & J.F. Smyth

*Imperial Cancer Research Fund Medical Oncology Unit, Western General Hospital, Edinburgh, EH4 2XU, UK.*

We have extended our previous report (*Cancer Res.,* **46:**

4916, 1986) showing that human alpha-2 interferon (IFN) can enhance the activity of certain cytotoxic agents (cyclophosphamide, ifosfamide and CDDP) in non-small cell lung cancer to other classes of anti-cancer drugs and other tumours. Doxorubicin, etoposide, vindesine, two platinum analogues and a new nitrosourea have been tested in combination with low doses of IFN in a squamous bronchial carcinoma xenograft. Although this tumour was generally resistant to most drugs, in all cases increased activity was seen with the combination compared with the drug given alone. IFN as a single agent had no effect. No such interactions were seen in two small cell xenografts with IFN in combination with four different drugs.

Using information from experiments in the xenograft model assessing different dosing schedules, we have undertaken a pilot clinical study of IFN and CDDP in patients with non-small cell lung cancer. Preliminary results from this study will be presented.

### The value of tumour markers in lung cancer

S.A. Gomm[1], B.G. Keevil[2] & N.T. Thatcher[1]

[1]*Department of Chest Medicine,* [2]*Department of Chemical Pathology, Wythenshawe Hospital, Manchester, M23 9LT, UK.*

The pre-treatment serum levels of neuron specific enolase (NSE), phosphohexose isomerase (PHI) and circulating immune complexes (CC) as tumour markers were compared to measurements of standard haematology and biochemical indices as an aid to the differentiation of tumour cell type, disease extent, response to therapy and survival in 76 patients with lung cancer. There were 57 males and 19 females, median age 59 years (range 31 to 78 years), 42 patients had small cell (SCLC) and 31 patients had non-small cell lung cancer (NSCLC) and 3 were of unknown histology. We observed elevated ($NSE > 12.5\,ng\,ml^{-1}$, $PHI > 120\,IU\,l^{-1}$, $CC > 55\,mg\,l^{-1}$) levels in 52.6% of cases for NSE, 85.5% for PHI and 47.6% for CC. NSE was significantly elevated in 61% of patients with SCLC compared to 40.6% with NSCLC ($P<0.005$), but there was no significant difference between limited and extensive disease in SCLC. CC levels were significantly raised in NSCLC (71.8%) compared to 31.7% in SCLC ($P<0.05$). Serum albumin levels were decreased significantly ($<30\,g\,l^{-1}$) in NSCLC, 46.9% compared to 14.6% in SCLC ($P<0.005$). Overall pre-treatment values of NSE, PHI and CC were not related to the clinical response to therapy, however, patients with an elevated GGT, AP or a low serum albumin were non- or partial-responders. Non-responders had 7 or more abnormal haematology and biochemical indices. Median survival was 10 months for the 76 patients and was not influenced by the levels of individual tumour markers, but was significantly shortened (7 months to 8 months, $P<0.00001$) when more than 4 abnormal indices were present.

### Serum C-reactive protein concentrations during chemotherapy for small cell carcinoma of the lung. A prospective serial study in 48 patients

C.R.K. Hind[1], & E. Lockey[2]

[1]*Royal Liverpool Hospital, Liverpool, L7 8XP; and* [2]*London Chest Hospital, London, E2 9JX, UK.*

Problems in patients with small cell carcinoma of the lung (SCCL) include assessment of tumour response to chemotherapy and early recognition of recurrence. No single laboratory abnormality is specific for SCCL, so these assessments are made by radiological techniques which are expensive and of variable accuracy. Serum C-reactive protein (CRP) levels rise nonspecifically in response to tissue injury and retrospective studies have suggested that these levels closely reflect tumour mass in carcinoma of the breast, colon and stomach. Serial serum CRP measurements were therefore performed prospectively in 48 patients undergoing chemotherapy for SCCL. No correlation between initial CRP levels and extent of disease was possible since several patients had just undergone biopsy procedures or had other conditions, e.g. infection) which in their own right result in an elevated CRP level. However, 2–4 weeks after the first chemotherapy the serum CRP was normal in 26/39 cases (of which 7 relapsed within 3 months) and raised in 13/39 (2 relapsed within 3 months). CRP sensitivity (proportion with raised levels) for extrathoracic relapse (liver, bone, brain) was 71% (10/14), and for intrathoracic relapse 24% (4/17). A raised CRP level only preceded clinically detectable relapse in 7% (1/14), though predicted unresponsiveness to relapse chemotherapy in all three cases. In conclusion, this study suggests that serum CRP levels only provide an accurate assessment of tumour response to relapse chemotherapy and not to initial treatment.

### The detection of occult bone marrow metastases from small cell lung cancer (SCLC) using immunocytochemistry

P.R. Kelsey

*Department of Haematology, Manchester Royal Infirmary, Manchester, UK.*

The application of immunocytochemistry to routine bone marrow smears as an aid to the detection of certain types of metastatic bone marrow involvement has been described (Fhosh *et al., Br. J. Haematol.* **61,** 21, 1985) and it can be of particular value in the identification of isolated suspicious cells. We set out to discover whether the sensitivity of bone marrow aspirates in the detection of SCLC could be increased by supplementing routine Romanovsky staining with immunoalkaline phosphatase staining using suitable primary antibodies. Two monoclonal antibodies were used: IJ13A, an antibody reactive with antigens expressed on cells of neural crest origin and LP34, reactive with cytokeratin. Marrow was aspirated from a single site on the posterior iliac crest and 6 smears prepared. Two smears were stained with May Grunewald Giemsa and 2 with each of the monoclonal antibodies mentioned above using a three stage immunoalkaline phosphatase technique. Material from 40 patients was examined and in 5 there was evidence of obvious bone marrow involvement on the Romanovsky stained slides. In all 5 of these cases the tumour cells stained strongly with UJ13A, but in only 3 were they stained by LP34. In no case did immunocytochemistry detect occult tumour cells in those marrows which had appeared morphologically normal. Whilst the strong positivity in the morphologically obvious cases attests to the efficacy of the technique, it does not seem likely that this approach can improve the sensitivity of routine bone marrow aspiration in the detection of occult involvement of SCLC.

### Immunocytochemical (IC) detection of marrow metastases in small cell lung cancer (SCLC) with possible clinical implications

R.C.F. Leonard, F.G. Hay, L.W. Adams, M.A. Cornbleet & J.F. Smyth

*ICRF Medical Oncology Unit, Department of Clinical Oncology, Western General Hospital, Edinburgh, UK.*

In a consecutive series of 27 patients with SCLC we compared 3 techniques of detecting tumour cells in marrow: (1) conventional smear and trephine, (2) haematological cytospin, and (3) multispot slides after Ficoll Hypaque separation. Techniques (2) and (3) were based on a panel of monoclonals against epithelial, neuroendocrine and 'lung cancer-associated' antigens selected by screening SCLC cell lines and clinical cancers as well as normal bone marrow. In 18 patients pretreatment, cytospin and smear/trephine were equivalent, detecting 4/13 cases with otherwise extensive, and 0/5 with otherwise limited SCLC. Multispot examinations however showed 11/12 extensive and 4/5 limited disease to have marrow metastases. Serum-free cell culture of marrow was feasible in 22 cases and all 'multispot positive' cases grew SCLC-like cells from marrow from 4–36+ weeks. Post-treatment, 9 patients in clinical CR were reassessed for chemo-intensification including autologous bone marrow rescue. 7/9 had marrow involvement by IC of whom 5 subsequently relapsed at metastatic sites 2–6 months later, regardless of further treatment.

### C-reactive protein in bronchial carcinoma

S. Mallya[1], L. Copeland[1], R.J. Donnelly[2], R. Mallya[1], & J.G. Williams[1]

[1]*Halton General Hospital, Runcorn; and* [2]*Broadgreen Hospital, Liverpool, UK.*

C-reactive protein (CRP) is an acute phase reactant present in trace amounts in the plasma. Its level rises in disorders associated with infection, infarction and malignancy. We wished to determine if its level was a good screening test for bronchial carcinoma and whether it could discriminate between localised and metastatic disease. Fifty-three patients with bronchial carcinoma were studied at initial presentation. Bony and/or hepatic metastases were detected clinically and radiologically in 23 of these patients. The control group comprised 40 patients with benign nodules, effusions or haemoptysis. C-reactive protein measurements were performed using the EMIT assay (Syva Co.). Median levels were calculated for each group and inter-group differences were calculated by Wilcoxon's Rank Sum Test. CRP levels are not a reliable screening test for bronchial carcinoma as 11 out of 31 patients with localised disease had normal values. Patients with localised disease had a significantly higher level than the control group ($P<0.01$). Patients with hepatic or bony metastases had a significantly higher level than those with localised disease ($P<0.001$). We conclude that CRP measurements are reliable indicators of hepatic/bony metastases in bronchial carcinoma but are not useful in detecting localised lesions.

### Characterisation of small-cell lung cancer cell lines established from both chemosensitive and chemoresistant tumours

R. Milroy, J. Plumb, R. Adamson, S. Banham, & S.B. Kaye

*Departments of Medical Oncology, University of Glasgow and Respiratory Medicine and Pathology, Royal Infirmary, Glasgow, UK.*

Small-cell lung cancer is usually chemoresponsive at outset. However, following complete response to treatment relapse is a frequent occurrence. Such relapse tumour is often resistant to further chemotherapy. Comparison of cell lines derived from pre-treatment and from relapse tumours may give an insight into the mechanisms of drug resistance. Thus, we have successfully established 5 small-cell lung cancer cell lines *in vitro* from 17 biopsies (1/7 bronchial biopsies taken via the fibre-optic bronchoscope and 4/10 metastasis).

Three pre-treatment lines were established from: (a) an endobronchial biopsy from a subsequently chemoresistant patient (LS106), (b) a metastatic node deposit from a subsequently chemosensitive patient (LS111) and (c) a cutaneous metastasis from an untreated patient (LS111). Two further cell lines of biopsies obtained from a patient who relapsed twice following chemotherapy have been established following passage in athymic nude mice.

These lines grow as floating aggregates of cells in RPM1 1640 culture medium supplemented with FBS (2½%) selenium, insulin and transferrin.

A sub-line of LS112 grows as a monolayer culture in Waymouths medium containing FBS (10%). The lines have been characterised by pathological and immunocytochemical examination. The cells express both dopa decarboxylase and creatine kinase BB iosoenzyme activities. LS112 monolayer also expresses creatine kinase MM isoenzyme activity. We are currently evaluating a suitable chemosensitivity assay for these cell lines to further investigate the question of drug resistance.

### Problems associated with chemosensitivity measurements of non-adherent small-cell lung cancer cell lines

J.A. Plumb, R. Milroy & S.B. Kaye

*Department of Medical Oncology, University of Glasgow, Glasgow, G12 9LX, UK.*

Drug resistance is a major problem encountered in the treatment of lung cancer. In order to study the mechanisms of drug resistance we have established a number of small-cell lung cancer cell lines. These tumours grow mainly as floating aggregates *in vitro*, many of which do not disrupt into single cell suspensions. Thus, conventional chemosensitivity assays are invalid. Recently, an assay based on the reduction of a tetrazolium dye (MTT) by live, but not dead cells has been described. We have characterised and improved this assay to allow chemosensitivity estimations of both adherent and non-adherent lung cancer cell lines.

The assay assumes a linear relationship between cell number and the amount of dye reduced by the cells. We have shown that this assumption is valid only if the MTT concentration is varied depending on the cell line used. This assay can also be used for many non-adherent cell lines. However, for some early passage small-cell lung cancer cell lines, the results suggest that drug exposure results only in a growth delay. Hence, spheroid growth delay measurements may be of more value for such cell lines.

### Assessment of lung cancer; non-small cell lung cancer

### An evaluation of the palliative role of radiotherapy in inoperable carcinoma of the bronchus

T.M. Collins, D.V. Ash, H.J. Close & J. Thorogood

*Cookridge Hospital, Leeds, UK.*

96 patients with inoperable carcinoma of the bronchus were entered into a prospective study of the effectiveness of palliative radiotherapy. Major symptoms were well controlled at 3 months (haemoptysis 88%, cough 58%, dyspnoea 53%) and 6 months follow up but dysphagia and extra thoracic systemic symptoms responded poorly. In spite of symptomatic improvement and objective response on X-

ray there was no significant effect on overall performance status. Some patients felt that the treatment had helped them even though performance status and symptoms were recorded as being worse. Side effects of dysphagia and tiredness occurred in 81% of patients but were classed as mild in 40% and 47% respectively and lasted less than 4 weeks in 86%.

There was no correlation between radiotherapy dose or radiological response and symptom control. The median survival of the group as a whole was 38 weeks. 14% of patients were dead within 3 months and were unlikely to have benefitted from therapy. Better identification of this group may spare them unnecessary treatment.

**Carcinoma of bronchus 1976–1983: A report of the Thoracic Group of the Yorkshire Regional Cancer Organisation**

C.K. Connolly[1], W.G. Jones[2], J. Thorogood[3] & C. Head

[1]*Friarage Hospital, Northallerton, North Yorkshire;* [2]*University Department of Radiotherapy; and* [3]*YRCO, Cookridge Hospital, Leeds, UK.*

20,155 cases of bronchial cancer with survival data were registered in Yorkshire between 1976 and 1983. During this period there was a progressive rise in mean age (65.9 to 68.2 years) and in the rate of histological confirmation (45.4 to 58.4%). The annual number of confirmed oat cell cancers rose from 209 (8.9%) to 308 (12.3%) and squamous case from 484 (20.5%) to 712 (28.4%). There was a doubling of histological confirmation of oat cell cancer in patients over 60 and of squamous cancers in patients over 70. Radical operations increased from 174 (7.4%) to 241 (9.6%). The number given chemotherapy without histological diagnosis fell from 98 (7.6%) to 32 (3.1%). No improvement in survival was seen for squamous carcinoma or in those treated with radical surgery or radiotherapy. Short term prognosis was improved in oat cell patients under 60 years given chemotherapy ($P=0.0002$). For all bronchial cancers, 2-year survival rose from 6.8% to 9.5% with significant improvement in subjects over 70 ($P=0.0123$). As little therapeutic advance was made, improved survival, especially in patients over 70 is more likely to be due to increased diagnostic activity and patient selection.

**Mitomycin, ifosfamide and *cis*-platin in non-small cell lung cancer**

M.H. Cullen, A.D. Chetiyawardana, R. Joshi[1] & C.M. Woodroffe

*Queen Elizabeth Hospital, Birmingham and* [1]*Manor Hospital, Walsall, UK.*

Mitomycin, ifosfamide and *cis*-platin are three of the most active single agents in the chemotherapy of non-small cell lung cancer. We have combined them in a 24 h schedule (MIC) for a phase 2 study in patients with inoperable non-small cell lung cancer. The regime consists of: mitomycin 6 mg m$^{-2}$ i.v. bolus, ifosfamide 3 g m$^{-2}$ i.v. infusion over 3 h and *cis*-platin 50 mg m$^{-2}$ i.v. infusion over 1 h. Mesna is given with the ifosfamide infusion at a dose of 1 g m$^{-2}$ and a further 500 mg m$^{-2}$ bolus is given 3 h later followed by a 6 h infusion (500 mg m$^{-2}$). The anti-emetic regime consists of lorazepam 2 mg m$^{-2}$ dexamethasone 8 mg m$^{-2}$ and metoclopramide 1 mg kg$^{-1}$ i.v. 30 min prior to chemotherapy, metoclopramide 9 mg kg$^{-1}$ over 24 h and further doses of dexamethasone 4 mg m$^{-2}$ i.v. 4 hourly.

Forty ambulatory (WHO performance status 0,1 or 2) patients with inoperable limited (LD) or extensive stage (ED) disease have entered this study, and 35 are evaluable for response. Fifteen patients have achieved partial remission (43%) and 5 have achieved complete remission (14%) as assessed radiologically. The overall response rate is thus 57%. There have been 14/20 responses in LD (70%) and 6/15 in ED (40%). Thirteen patients have experienced an improvement in WHO performance status rating. Although well tolerated in the majority of patients, the principal toxicity has been vomiting which was severe (WHO 3/4) in 6 patients.

MIC is clearly among the most active combinations in non-small cell lung cancer and will now be tested in a randomized trial against no chemotherapy.

**Antibody guided targetting of breast cancer and non-small cell lung cancer using indium-111 labelled HMFG1-F(ab′)$_2$ fragments**

H. Kalofonos, J. Taylor-Papadimitriou, J.P. Lavender & A.A. Epenetos.

*ICRF Oncology Group, Royal Postgraduate Medical School, Hammersmith Hospital, Du Cane Road, London, W12 0HS and Imperial Cancer Research Fund, Lincolns Inn Fields, London, WC2A 3PX, UK.*

F(ab′)$_2$ fragments of tumour associated monoclonal antibody HMFG1 were used for radioimmunolocalisation of primary and metastatic breast and non-small cell lung cancer.

The antibody was conjugated with DTPA and labelled with Indium-111. Radio-labelled antibody was shown to be stable *in vitro* and *in vivo* and there was no significant loss of immunoreactivity.

10 patients with breast and 11 with lung cancer were studied by radioimmunoscintigraphy after i.v. administration of indium-111 In-labelled HMFG1 F(ab′)$_2$.

Successful localisation was observed in all primary and in the majority of metastatic lesions. No significant uptake was observed in normal organs except for liver which accumulated ~20% of the injected amount. No toxicity was encountered and no human anti-mouse response was detected in any of the patients even after repeated administration.

**Clinical results of pulmonary resections in carcinoma of the bronchus**

D. Kaplan, D. Muehrcke, & R.J. Donnelly

*Regional Cardio Thoracic Unit, Broadgreen Hospital, Liverpool, UK.*

Between January 1980 and December 1986, 564 patients underwent pulmonary resection for malignant disease. Automatic stapling devices were used to close the bronchus in all patients. The operative mortality was 4.9% (3.2% in patients undergoing lobectomy and 8.3% in those undergoing pneumonectomy). The complication rate was 17.3%. The post-pneumonectomy incidence of fistula was 2.8% within three months of surgery and 4.6% overall. Stapled closure of one or more hilar vessels was employed in 153 patients. Pulmonary resection can be performed with acceptably low morbidity and mortality.

**Survival after radiotherapy for lung cancer**

L.M. Matheson, S. Capewell, M.F. Sudlow & G.A. Newaishy
*on behalf of the Edinburgh Lung Cancer Group*

*Department of Clinical Oncology, Western General Hospital, Edinburgh, UK.*

The Edinburgh Lung Cancer Group (ELCG) prospectively registered 2586 new cases of lung cancer during 1981–84. Initial treatment options included thoracic irradiation (31%), surgery (19%) and chemotherapy (12%); 38% received symptomatic therapy only.

In 1981, 233 patients with non-small cell lung cancer (NSCLC) were treated with primary thoracic irradiation. Radical radiotherapy (radical RT, central dose 45–57 Gy) was given to 41 (17%) patients of whom 32 had positive histology; 192 received palliative irradiation (palliative RT: 18–30 Gy). Compared with the palliative group, the radically treated patients were younger (76% *vs.* 57% < 70 years; $P<0.02$), fitter (Karnofsky index <80 in 79% *vs.* 49%; $P<0.001$) and had less extensive disease.

After radical RT for histologically proven NSCLC, local control (radiological CR + PR) was achieved in 25/32 (78%) and was a prognostic factor for survival; 4 year survival was 12.5% (4/32). After palliative RT, 7/192 survived 4 years.

These data suggest that radical thoracic, irradiation achieves good local control of disease and may confer a survival benefit in selected patients with inoperable NSCLC.

### Central nervous system (CNS) surveillance with computerised tomography (CT) and magnetic resonance (MR) imaging in small cell lung cancer (SCLC): A comparison of imaging modalities.

T.J. Perren, I.E. Smith, G. Cherryman, J. Husband, M. Williams & C. Heron

*Lung Unit and Department of Radiology, Royal Marsden Hospital, Sutton, Surrey, UK.*

49 patients with SCLC entering a 3 monthly CT scan surveillance study designed to detect CNS metastases whilst asymptomatic, also had MR within 1 week of each CT scan as part of a second study to compare the 2 imaging modalities. 2 additional patients with known CNS metastases were also scanned; 1 at the time of recurrence and the other 3 months after cranial radiotherapy. A Siemens Magnetom 2 tesla (T) unit operating at 1.5T was used. 7mm axial sections were obtained at 3.5mm gaps using a $T_2$-weighted sequence with TR 2.7sec and TE of 30 + 90 m sec. Abnormal scans were repeated using $T_1$-weighted sequences. 16 patients also received 0.4 mg kg$^{-1}$ of gadolinium DTPA IV contrast medium (Schering UK), post contrast scans were obtained using $T_1$-weighted sequences.

8 patients developed CNS metastases and 1 had recurrent metastatic disease; in 8 of these 9 patients lesions were identified with both CT and MR, and in the 9th pt only on MR. Of the 8 patients with positive CT and MR, MR was considered superior to CT in 2 and equivalent to CT in 3. The remaining 3 patients had multiple lesions, some better seen with MR and some better seen with CT. 3 patients with metastases were rescanned after the injection of gadolinium DTPA and in all instances metastases enhanced strongly with contrast allowing lesions to be seen with greater clarity and confidence. Additional lesions were also seen on post contrast studies. 1 patient scanned 3 months after radiotherapy to a CNS metastasis showed strong gadolinium enhancement of the residual lesion, although the CT had returned to normal; and in another patient, rescanned 2 months after cranial radiotherapy, the residual lesion was better seen on gadolinium MR than on CT.

These preliminary results suggest MR may be useful in CNS surveillance of patients with SCLC.

### High dose rate intracavitary radiation in the treatment of carcinoma of the lung

C.G. Rowland & K.M. Pagliero

*Departments of Radiation/Oncology and Thoracic Surgery, Royal Devon and Exeter Hospital (Wonford), Exeter, Devon, EX2 5DW, UK.*

Endobronchial brachytherapy has been performed up to 30 years in a limited number of centres; mainly on an anecdotal basis. Unfortunately, due to the limitations and size of equipment available only a limited number of tracheal and main bronchial tumours have been accessible by these techniques. Successful treatment of more peripheral tumours with more sophisticated, safe after loading equipment has recently been reported from North America. Patients treated include those who have not only primary tumours but also recurrences following previous surgery, radiation and chemotherapy. In conjunction with the Dutch firm, Nucletron, a high dose rate micro-selectron has been developed to allow the passage of a small, intense iridium source of up to 10 curies activity. The treatment catheter which is 2 mm in diameter can either be wire guided or directly placed through the side channel of the more modern bronchoscopes. It can also be placed transcutaneously, and depending upon the source activity the treatment time can be as short as 10–15 min.

It is important that the effect of intracavitary radiation alone in the treatment of lung cancer should be documented as most other studies have involved multi-modality treatment. A clinical trial supported by the South Western Regional Health Authority has been set up.

### A numerical discriminant applied to prediction of survival in inoperable carcinoma of the bronchus

J. Thorogood, T.M. Collins, D.V. Ash & H.J. Close

*Cookridge Hospital, Leeds, UK.*

An evaluation of the palliative role of radiotherapy in inoperable lung cancer revealed that 16 out of 96 patients studied (17%) were dead within 3 months of treatment and were unlikely to have benefited from therapy. A statistical discriminant approach was used to identify a suitable index to predict survival to 3 months. This index comprised weight loss in the previous 6 months, performance status, extent of disease and blood lymphocyte count.

A simple tree diagram was developed for clinicians to follow through for a given patient, to arrive at one of 16 discrete probabilities of that patient being alive 3 months later. No calculations are required for its use and it is based on the index, using four factors only. The survival probabilities ranged from over 0.95 for 3 or 4 factors in the good prognosis category, to less than 0.20 with 3 or 4 factors in the poor category.

When applied to a prospectively collected set of 80 patients, the index achieved an accuracy of 97% in predicting those who would survive for longer than 3 months. It achieved a low accuracy of 26% in those dying before 3 months because these cases presented with 2 good factors and 2 poor.

### Sequential combination chemotherapy and radiotherapy in locally advanced non small cell carcinoma of the bronchus

S.W. Watkin, R.D. Errington, J.A. Green & H.M. Warenius

*CRC Department of Radiation Oncology, Clatterbridge Hospital, Bebington, Wirral, Merseyside, UK.*

The feasibility, effectiveness and toxicity of a new 4 drug intensive combination chemotherapy regime has been assessed. Sequential radical radiotherapy was given to all patients after completion of chemotherapy. Response, local control and survival following this strategy have been evaluated in a group of patients with inoperable non-small cell lung cancer. 35 patients, 24 male, 11 female, aged 35–71 years, mean 56.2 years have been entered into the study. ECOG performance status was 0/1 in 25, 2/3 in 8 and 4 in 2. Histology was squamous carcinoma 21, adenocarcinoma 6, large cell 2, undifferentiated in 5 and there was 1 alveolar cell carcinoma. 15 patients were stage III M0 and 19 were stage III M1 in whom 8 had neck nodes as the only site of metastasis. Patients were given adriamycin 40 mg m$^{-2}$, vindesine 3 mg m$^{-2}$, ifosfamide 5 g m$^{-2}$ with mesna rescue, and *cis*-platin 60 mgm$^{-2}$. Four weeks after the last cycle of chemotherapy, radiotherapy to a total dose of 4800 cGy in 16 fractions was given to the primary tumour. 22 (63%) patients have received 3 or more courses of chemotherapy and 25 (71%) have received radiotherapy of whom 14 received wide field radiotherapy and bulk boost to the planned dose. Response to AVIP chemotherapy was CR 3 (9%), PR 9 (26%), SD 13 (37%), PD 4 (11%). There have been 2 early deaths (PD in 1, toxicity in 1) and 4 have not completed chemotherapy. Nadir blood counts in 94 cycles showed WBC $< 2.5 \times 10^9$ l$^{-1}$ in 32, Hb $< 10$ g l$^{-1}$ in 18 and platelets $< 100 \times 10^9$ l$^{-1}$ in 13. There were 3 neutropenic fevers. Median survival is 10 m and 12 m survival was 40%. Local relapse has occurred in 12 patients, 4 of whom had received DXT 48 Gy, and brain metastases occurred in 6. A further 6 patients have not relapsed. This strategy produces favourable response rates with acceptable toxicity.

### Small cell lung cancer; new drugs in lung cancer

### Pretreatment prognostic factors in 407 patients with small cell lung cancer and 134 patients with non small cell lung cancer

H. Anderson[1], T. Cerney[1], R. Swindell[2] & N. Thatcher[1]

[1]*CRC Department of Medical Oncology; and* [2]*Department of Medical Statistics, Christie Hospital, Manchester, UK.*

In 407 patients with small cell lung cancer (SCLC), 61 pretreatment variables were evaluated by Cox Multiple regression analysis to look at prognostic factors. All patients received 18 weeks of intensive chemotherapy and thoracic radiotherapy. 6 variables were found to be the most important and other factors added no additional prognostic information.

The prognostic factors were LDH ($P=0.0001$), stage ($P=0.001$), serum sodium ($P=0.0009$), Karnofsky performance ($P=0.0121$), alkaline phosphatase ($P=0.0186$), serum bicarbonate ($P=0.0321$). A simple scoring system was established and analysis distinguished 3 prognostic groups, the best group containing all the 2+ year survivors and 19% of these had extensive stage disease.

In 134 patients with non small cell lung cancer (NSCLC), 43 pretreatment variables were evaluated by Cox multiple regression analysis to assess the effect on prognosis. All the patients had received ifosfamide chemotherapy for 12–18 weeks and some had also received some cyclophosphamide. Karnofsky performance was the variable with the most prognostic importance ($P=0.0001$). The serum chloride was of border significance ($P=0.04$). A scoring system using Karnofsky performance and chloride divided patients into prognostic groups and the best group also contained patients with extensive stage disease.

Patient selection for chemotherapy regimens based on stage alone may not be adequate.

### Small cell lung cancer – therapy with i.v. bolus ifosfamide and 8 day oral VP16

H. Anderson[1], M.J. Lind[1], K. Carroll[2] & N. Thatcher[1]

[1]*CRC Department of Medical Oncology, Christie Hospital, Manchester; and* [2]*Department of Chest Medicine, Wythenshawe Hospital, Manchester, UK.*

From May 1986, 46 patients with small cell lung cancer have received therapy with ifosfamide 1.5 gm m$^{-2}$ mixed with mesna 1.5 gm m$^{-2}$ as a short i.v. infusion in 500 ml normal saline over 30 min. Patients received 200 mg oral mesna at 4 and 8 h after therapy and oral VP16 100 mg daily for 8 days. Patients stayed in hospital overnight the day of chemotherapy. Cycles were repeated at 3 weekly intervals if the WCC was $> 3.0 \times 10^9$ l$^{-1}$ and platelets $> 100 \times 10^9$ l$^{-1}$. The aim was to give 6 courses of CT and reassess. 27 patients (16 male: 11 female) have now completed therapy. The median age was 63 (45–73 years) 10 patients had limited stage (LS) and 17 extensive stage (ES) disease (9 liver, 5 bone, 3 nodal). The median KP before treatment was 60 (range 40–80) and MRC respiratory score 3 (range 1–5).

A median of 6 courses has been given (range 2–6) and the 10 LS patients have received thoracic irradiation. Toxicity was little. The median WHO grade for haematological toxicity was 1 (range 0–3). Nine patients had no haematological toxicity. Non-haematological toxicity median WHO grade was 1 (range 0–3), and usually was transient nausea. Blood transfusions were given to 8 (30%) patients and i.v. antibiotics to 6 (22%).

The median KP post treatment was 70 (range 0–100), and RS 2 (range 1–5). The overall response rate was 33/27 (48%) with 6 (22%) complete remissions. The median survival was 6/12 for ES and 7/12 for LS disease. This low dose non toxic therapy is inferior to our other regimens and has been discontinued.

### Chemotherapy plus adjuvant surgery for local small cell lung cancer

G.F.A. Benfield[1], H.R. Matthews[2], D.C.T. Watson[2], F.J. Collins[2] & M.H. Cullen[1]

[1]*WMCRC Clinical Trials Unit, Queen Elizabeth Medical Centre, Birmingham, B12 2TH; and* [2]*Department of Thoracic Surgery, East Birmingham Hospital, Birmingham, B9 5ST, UK.*

Chemotherapy is effective radical treatment for small cell lung cancer (SCLC), but relapse is usual with up to 85% of tumours recurring at the primary site. Surgery is an effective debulking agent, but < 10% of tumours are amenable to this approach because of the early metastasis of SCLC. An alternative approach would be to combine these 2 modalities to derive their respective benefits.

Nine patients with local Stage II (T2N1M0) or III (T1N2M0) SCLC received combination chemotherapy – 2

courses of cyclophosphamide, doxorubicin, etoposide, vincristine and methotrexate – followed by adjuvant surgery, to assess the effectiveness of such a regimen in improving operability, preventing local relapse and extending survival. The regimen was well-tolerated and prevented local relapse. However, the median time to recurrence of disease was 10 months and the median survival time 12 months, results which are similar to those achieved with chemotherapy and radiotherapy. Distant metastases, particularly in the brain, occurred predictably indicating that successful adjuvant surgery, despite preventing local relapse, does not appear to afford additional survival benefit.

### A randomised trial of short courses of intravenous (i.v.) chemotherapy *versus* oral (p.o.) out-patient chemotherapy for small cell lung cancer (SCLC)

B.J. Cantwell[1], A.L. Harris[1], J.M. Bozzino[1], P. Corris[2] & D. Veale[2], and the North East of England Lung Cancer Study Group

[1]*University Department of Clinical Oncology, Regional Radiotherapy Centre, Newcastle General Hospital; and* [2]*Department of Respiratory Medicine, Freeman Hospital, Newcastle upon Tyne, UK.*

A regional randomised trial compared i.v. chemotherapy *versus* oral chemotherapy for histologically or cytologically proven SCLC. Patients were randomised to receive either p.o. chemotherapy (chlorambucil 6 mg m$^{-2}$ daily for 10 days, procarbazine 150 mg daily for 10 days, prednisolone 20 mg daily for 10 days and VP16 300 mg daily for 3 days only) or i.v. chemotherapy (adriamycin 40 mg m$^{-2}$, vincristine 2 mg, VP16 100 mg m$^{-2}$, all i.v. on day 1, with oral VP 300 mg on days 2 and 3 – course 1, and for courses 2, 3 and 4, adriamycin 30 mg m$^{-2}$, vincristine 2 mg, VP16 200 mg m$^{-2}$ i.v., plus ifosfamide 5 g m$^{-2}$ and mesna by 24 h i.v. infusion). Both p.o. and i.v. cycles were repeated every 21 days to a total of 4 courses. Patients with good response to chemotherapy in either arm were given consolidation mediastinal and prophylactic cranial radiotherapy. 110 patients received i.v. and 100 p.o. chemotherapy and both arms were matched for sex, age and performance status. Response rates were: p.o. (CR) = 22%, i.v. CR = 22%, p.o. PR = 45%, i.v. PR = 50%, in limited (L) stage disease. In extensive (E) stage disease i.v. chemotherapy produced a greater PR rate (p.o. PR = 27%, i.v. PR = 59%; $P<0.01$). At a median follow up of 96 weeks L stage patients had better survival than E stage patients ($P<0.01$), log rank test, but there were no differences between p.o. *vs.* i.v. treatments overall or within L stage disease. Patients with E disease had better survival if given i.v. chemotherapy ($P<0.05$). There were 8% toxic deaths in i.v. and 3% in p.o. patients. Short courses of p.o. out-patient chemotherapy confer equal survival benefit as the more expensive i.v. chemotherapy for L stage SCLC but i.v. treatment is better for E stage disease.

### Late intensification with high dose melphalan (HDM) + autologous bone marrow rescue (ABMR) in small cell lung cancer (SCLC): An analysis of patterns of failure

M.A. Cornbleet, A. Gregor, R.C.F. Leonard & J.F. Smyth

*Department of Clinical Oncology, Western General Hospital, Edinburgh, UK.*

'Complete' remission (CR) rates of 30–50% are regularly reported in patients with limited stage (LS) SCLC but long-term disease control is infrequent. Patients with good performance status and LS in bronchoscopically confirmed CR (BCCR) were offered late intensification of therapy comprising HDM (140 mg m$^{-2}$) and ABMR, followed by radiotherapy (RT) to the primary site (4500 cGy in 20 fractions) and prophylactic cranial irradiation (PCI). 23/71 (32%) achieved BCCR after a 12-week induction regimen comprising methotrexate, cyclophosphamide, etoposide 3 adriamycin × 4. 11 declined HDM and received the same RT. HDM + AMBR was well tolerated, being associated with acute nausea and vomiting, predictable myelosuppression and neutropenic PUO (4 patients – total febrile days = 13) with no treatment-related mortality. Median disease-free (DFS) and overall survivals (OS) were: 10 & 12 mo after HDM and 15 & 17 mo without. Actuarial 2-year survival was 0% and 21% respectively. 16/23 patients have relapsed; 8/11 without HDM, 8/12 with. Initial sites of relapse were: no HDM chest 5 (1 infield, 4 marginal), cervical nodes 1, liver 1, brain 1 (prior to PCI); with HDM – chest 4 (all infield), liver 3, brain 1 (prior to PCI). The 4 patients relapsing initially in the liver had fulminating courses; none survived >6 weeks. All 5 non-CNS distant relapses had the primary site controlled until death. We conclude that: (a) 12 weeks' systemic therapy is insufficient for this good prognosis subset of patients; (2) local control remains a major problem; (3) possible occult reinfusion of tumour cells indicates the need for improved methods of detection; (4) although HDM given as late intensification in this dose and schedule does not significantly enhance DFS or OS, higher doses and different schedules are feasible and are currently under investigation.

### Testing new drugs in untreated small cell cancer may prejudice the efficacy of standard treatment: A phase II study of oral idarubicin in extensive disease

M.H. Cullen, S.R. Smith, G.F.A. Benfield & C.M. Woodroffe

*Queen Elizabeth Hospital, Birmingham, B15 2TH, UK.*

The results of standard chemotherapy treatment in small cell cancer are poor with virtually no long term survivors. However a number of chemotherapeutic agents, in combination, can provide effective short term palliation and extension of survival. Conventionally, new drugs are tested in patients who have failed on standard treatment and thus, by definition, have multi-drug resistant tumours. It is quite possible that potentially valuable agents will not be identified in this setting. Thus a number of investigators have tested new drugs in previously untreated cases, reverting to standard treatment in those who fail to respond. We have evaluated the orally active anthracycline idarubicin (40 mg m$^{-2}$ in divided doses over 24 h) in 22 patients with extensive stage small cell lung cancer, and report the results of subsequent i.v. therapy ('CVE'-cyclophosphamide 1000 mg m$^{-2}$, vincristine 1 mg m$^{-2}$, etoposide 120 mg m$^{-2}$ i.v. day 1; etoposide 250 mg m$^{-2}$, orally in divided doses on day 2), as well as those of the phase II study.

Of 22 treated patients 3 (14%) responded with 2 complete remissions. Patients failing to respond promptly, and relapse cases were treated with CVE. Seven patients did not receive CVE for the following reasons: early death 4, early CNS disease 2, refusal 1. Fifteen cases did receive CVE. Of 13 idarubicin failures 9 progressed on CVE, 3 achieved PR and 1 CR. Two idarubicin responders who had CVE achieved PR and CR. The median survival (MS) of all 22 patients is 136 days. For those with WHO performance scores of 0 or 1 the MS is 189 days and for the rest it is 80 days. Despite a strictly applied policy of instituting standard i.v. therapy promptly in static or progressive disease, these results are inferior to those seen in our centre with standard treatment from the start, in extensive small cell cancer, especially in those with poorer performance status.

We believe that patients with extensive small cell cancer often require a rapid and more guaranteed response to their first treatment and may not be suitable for experimental therapy.

### Prognostic factors in patients with small cell lung cancer (SCLC)

H.M. Earl[1], R.L. Souhami[1], C.M. Ash[1], S.G. Spiro[2], D. Geddes[3], P.G. Harper[4] & J.S. Tobias[1]

[1]*Department of Radiotherapy and Oncology, University College Hospital, London, WC1E 6AU;* [2]*Brompton Hospital, London, SW3 6HP;* [3]*London Chest Hospital, E2 9JX; and* [4]*Guy's Hospital, London, SE1 9RT, UK.*

Analysis of pretreatment variables in 371 patients with SCLC treated with identical chemotherapy in the context of a large prospectively randomised clinical trial, demonstrated that prognosis was strongly correlated with initial performance status, plasma sodium and albumin, and plasma alkaline phosphatase (Souhami *et al., Cancer Res.*, **45**, 2878, 1985). Using these parameters 3 prognostic groups were defined. These prognostic factors were then analysed in 613 patients with SCLC who were included in a prospectively randomised trial of chemotherapy duration. The prognostic factors were confirmed, on this new data set, to be predictive of survival. Plasma alkaline phosphatase at diagnosis was predictive for early death, and performance status for both short and long term survival. Long survivors were predominantly patients with limited disease in the good prognostic category, whilst the majority of patients dying within 21 days were in the poor prognostic category. Analysis of the change in these parameters in response to treatment showed that clinical response was associated with improved survival, even in the bad prognostic category. Analysis of prognostic factors at diagnosis may not only provide guidelines for selecting patients suitable for intensive treatment, but also for identifying patients who are at high risk of early death, and who require modifications of standard chemotherapy. Sequential analysis of change in prognostic factors in response to treatment allows an assessment of the value of continued chemotherapy in poor risk groups, and a definition of the criteria of response using biochemical markers.

### Prognostic factors in small cell lung cancer – the Edinburgh experience

S.G. Allan, J.F. Smyth, M.A. Cornbleet & R.C.F. Leonard

*Department of Clinical Oncology, Western General Hospital, Edinburgh, UK.*

Between 1980 and 1986, 297 small cell patients have been entered into treatment studies in the Edinburgh Medical Oncology Unit. 81 patients were treated between 1980 and 1982 with methotrexate, CCNU and cyclophosphamide with or without maintenance therapy (which itself had no survival impact). In subsequent trials 100 'good prognosis' patients were treated with methotrexate, cyclophosphamide and VP-16, and 116 'poor prognosis' with vindesine and VP-16. The presenting clinical, radiological and biochemical characteristics were documented and retrospectively analysed within each treatment category and then in all treatment categories combined with the aim of identifying prognostic indices. Applying Mantel Log Rank statistics on actuarial survival analysis, we found that the most important individual factors common to all treatment groups were respectively performance status, disease extent, age, white blood count and bone scan. Low haemoglobin ($<12\,g\,dl^{-1}$) and or low serum sodium ($<135\,m\,mol\,l^{-1}$) had minor negative influence on survival ($P=0.08$ and $P=0.06$ respectively) but all other parameters tested i.e. gender, blood platelets, serum LDH, alkaline phosphatase and albumin were of minor or no importance in the treatment groups analysed in isolation or combined. Multivariate analysis (Cox model) confirmed the adverse affect on prognosis only of increasing performance status, increasing white blood count and extensive disease especially when the latter incorporates both liver and bone or bone marrow.

### Sequential induction chemotherapy with late intensification in small cell lung cancer

J.A. Green, J. Hardman, R.D. Errington & H.M. Warenius

*CRC Radiation Oncology Unit, Clatterbridge Hospital, Wirral, Merseyside, UK.*

Seventy-two patients with small cell lung cancer (48 limited, 24 extensive disease) were entered on a protocol comprising induction with 4–6 cycles of cyclophosphamide $1\,g\,m^{-2}$, adriamycin $40\,mg\,m^{-2}$ and vincristine $1.4\,mg\,m^{-2}$ (CAV) every 21 days for 4–6 cycles. 33 patients achieving complete remission received intensification with 2 further cycles of ifosfamide $5\,g\,m^{-2}$, mesna $8\,g\,m^{-2}$, methotrexate $30\,mg\,m^{-2}$ and etoposide $100\,mg\,m^{-2}$/day for 3 days (IME). 6 of the 13 patients in partial remission following CAV achieved a further remission on IME (response rate 46%). The overall median survival was 8 months, and of the 33 patients in complete remission was 11.5 months, with 3 long term survivors at over 2 years. Median survival in the limited disease group was 12 months compared with 5.5 months in the extensive disease patients. There was no significant prolongation of survival seen in those patients receiving prophylactic cranial irradiation (10 patients) mediastinal irradiation (12), or those who had negative second bronchoscopy examination. This sequential 6-drug regime produces acceptable response and survival in small cell lung cancer, but does not produce a cohort of long term survivors.

### Contribution of thoracic irradiation to local control in small cell lung cancer (SCLC)

A. Gregor[1,2], S.W. Banham[1], M.A. Cornbleet[2], A.J. Dorward[1] & J.F. Smyth[2]

[1]*West of Scotland Lung Cancer Group, Glasgow; and* [2]*Department of Clinical Oncology, Western General Hospital, Edinburgh, UK.*

The contribution of improved local control to survival is difficult to assess in diseases where short survival time is determined by metastatic spread. In SCLC, a number of regimens of thoracic irradiation are used, often with conflicting results. In order to assess the contribution of conventionally fractionated, moderate dose (40 Gy in 3/52–45 Gy in 4/52) radiotherapy (RT), given to the presentation volume after a 12-week course of induction chemotherapy, we have analysed local control in 55 limited disease (LTD) patients achieving bronchoscopically confirmed complete remission (CR). Patients were treated in 2 concurrently running studies (1982–1984) in Glasgow (GL) and Edinburgh (ED). Prognostic features, staging and assessment of response were similar in both studies. Induction chemotherapy was previously described. No maintenance chemotherapy or late dose intensification was given. 44/95 (46%) of LTD patients in GL achieved CR, 23 were irradiated; 21 control patients from one participating centre did not receive RT due to physician's preference. 23/71 (32%) LTD patients in ED achieved CR, 12 subsequently received melphalan $140\,mg\,m^{-2}$ and are excluded from this study. The remaining 11 patients were irradiated. Local relapse occurred in 11/34

(32%) of irradiated patients and 15/21 (71%) controls ($P<0.005$). It was the sole site of failure in 6 (18%) patients receiving RT and 8 (38%) controls. Detailed analysis of primary site relapses is in progress. No significant survival difference was found between the irradiated and control group, with median survivals 14 & 12 months respectively. Conventional RT significantly reduces local recurrence rate but its impact on survival even in the best prognostic group of patients is minimal.

### Chemotherapy for the elderly with small cell lung cancer

A.M. Hendrick[1,3], M.J. Connolly[1,2], D.J. Hendrick[1], B.M.J. Cantwell[3] & A.L. Harris[3]

*Departments of [1]Chest Medicine; [2]Geriatrics; and [3]Clinical Oncology, Newcastle General Hospital, Newcastle upon Tyne, NE4 6BE, UK.*

Local figures suggest that one third of patients presenting with small cell lung cancer are over 70 years of age. As adverse effects from combination chemotherapy at this age may be increased, we have evaluated the risks against benefit in 32 such patients receiving short courses of one of two chemotherapy combinations. Seventeen patients were treated with a conventional i.v. regimen of adriamycin 40 mg m$^{-2}$, vincristine 2 mg and VP 16213 100 mg m$^{-2}$ followed by VP 16213 300 mg orally on days 2 and 3. Six patients also received ifosfamide. The other 15 received oral therapy with VP 16213 300 mg for 3 days and chlorambucil 100 mg + procarbazine 150 mg + prednisolone 20 mg daily for 10 days. Compliance with this treatment was excellent despite the 99 tablets per course. Treatment groups did not differ in respect to clinical parameters and response rates (complete and partial), being 71% for the i.v. and 86% for the oral therapy. In the i.v. group there were four sudden (presumed toxic) deaths within the period of the first two courses of therapy, and a fifth death due to recognised cardiotoxicity. In addition, non-fatal neutropenic fever occurred in five subjects and cardiotoxicity in one. By contrast, the only serious complication in the oral group was a case of non-fatal neutropenic fever. ($P<0.01$) Median survival was 6.5 m for both treatment groups with only two patients in each treatment group alive at one year. Thus survival is inferior to that achieved with younger patients. It appears that this i.v. regimen is unduly toxic in the elderly but that a similar and worthwhile survival benefit can be achieved with oral therapy with minimal toxicity.

### Ifosfamide, etoposide and adriamycin in the treatment of small cell lung cancer

M.J. Lind[1], H. Anderson[1], M. Bronchud[1], N. Thatcher[1] & R. Stout[2]

*[1]CRC Department of Medical Oncology, Christie Hospital; and [2]Department of Radiotherapy, Christie Hospital, Manchester, M20 9BX, UK.*

23 patients with intermediate prognosis small cell lung cancer were treated with ifosfamide 5 g m$^{-2}$, mesna 8 g m$^{-2}$, adriamycin 50 mg m$^{-2}$, and etoposide 120 mg m$^{-2}$ i.v. on day 1, etoposide 120 mg m$^{-2}$ i.v. on day 2 and a further 240 mg m$^{-2}$ of oral etoposide on day 3. A total of 6 courses were given to each patient. In addition 8 patients with limited stage disease were given mediastinal radiotherapy following chemotherapy. The median age was 59 years, 10 patients having limited stage disease and 13 extensive stage disease. So far 120 courses have been administered. Non haematological toxicity has been mild consisting mainly of nausea and vomiting. However, haematological toxicity has resulted in 14 courses having to be delayed because of myelosuppression, 10 patients experiencing ≧ WHO grade 4 leucopenia.

To date there have been 8 complete responses and 11 partial responses giving an overall response rate of 83%. To date 3 patients have died from lung cancer and there has been one toxic death. 16 of these 19 responses were seen after 3 courses. Treatment resulted in a marked improvement of the Karnofsky performance score. Before treatment only 6 patients had a KP≧80 as compared with 17 one month following chemotherapy. There was a similar improvement in the MRC respiratory score.

### Carboplatin (C), Ifosfamide (I), Etoposide (E) with mid course vincristine (V) and thoracic radiotherapy for limited stage small cell lung cancer (SCLC)

M.J. Lind[1], H. Anderson[1], D.B. Smith[1], R. Stout[2] & N. Thatcher[1]

*[1]CRC Department of Medical Oncology; and [2]Department of Radiotherapy, Christie Hospital, Manchester, M20 9BX, UK.*

Carboplatin has high single agent activity in SCLC but has considerable myelosuppression. The I+E combination is associated with only mild toxicity. Starting in September 1985, the three drugs were used in combination with V. Thoracic radiotherapy either by a single rotation treatment or 8F parallel pair was given at the end of chemotherapy (CT). The regimen used was C-300 mg m$^{-2}$ i.v. day 1; I-5 g m$^{-2}$ over 24 h with mesna i.v. day 1; E-120 mg m$^{-2}$ i.v. days 1, 2 and 240 mg m$^{-2}$ orally day 3, with mid course day 14, V-0.5 mg i.v. The courses were repeated every 28 days for a total of six. 42 patients have entered the study, the metastases were pleural effusion 21%, nodes 19%, mediastinum 62%, elevated alk.phosph. 29%, LDH 31% and other liver enzymes 43%. So far 222 courses have been given and 72% of patients have received all 6 courses. The CR rate is 50%, PR 31% and 69% of all patients responded by course two. The median survival is 13 months, range 1–18+. Brain relapse as the solitary site has occurred in 19% of patients. There were three treatment related deaths with leucopenia and 7% of courses have been delayed because of toxicity. Nadir counts were in the majority ≧grade 3 usually occurring on week 3 after CT. Rapid improvement in the Karnofsky score in 71% patients occurred with the CT despite considerable myelosuppression. Prophylactic brain irradiation may have been indicated for this group of patients.

### Carboplatin (Cp), VP16 (V) and ifosfamide (I) intensive chemotherapy (CT) for small cell lung carcinoma (SCLC)

T.J. Perren, I.E. Smith, M.J. Plant & J.R. Yarnold

*Lung Unit, Royal Marsden Hospital, Sutton, Surrey, UK.*

Cp+V has proved very active against SCLC (85% response) but with short duration. A more intensive full dose 3 drug combination of Cp 400 mg m$^{-2}$ i.v. day 1, V 100 mg m$^{-2}$ i.v. day 1–3, and I 5 g m$^{-2}$ day 1 (24 h infusion) with mesna, q 28 days × 6 courses has therefore been used in 37 fit (ECOG performance status 0–2) previously untreated SCLC patients. Limited disease (LD) patients also received hyperfractionated thoracic radiotherapy concurrently with the first 2 CT courses (15 Gy × 15 × 5 days × 2 courses). Prophylactic cranial irradiation (PCI) was offered to all LD patients achieving CR. 30 patients (19 males) are so far evaluable (>2 courses CT), median age 60 (30–70) yrs. Response is as follows:

| | Patients | CR | PR | Overall Response |
|---|---|---|---|---|
| LD | 16 | 10 (63%) | 5 | 15 (94%) |
| ED | 14 | 3 (21%) | 11 | 14 (100%) |

For LD patients median survival has not been reached, 1 year actuarial survival is 85%; for ED patients median survival is 8 months. 3 LD patients (19%) have had systemic relapse, and 3 asymptomatic CT scan CNS relapse before PCI; 9 ED patients (64%) have relapsed. Main toxicity was myelosuppression: 72% patients required dose reduction, 7% treatment delay, and 7% (2) had treatment deaths. Main toxicity details are as follows:

| | WHO Grade | | |
|---|---|---|---|
| | 0 | 1–3 | 3–4 |
| WBC suppression | 0 | 0 | 30 (100%) |
| Platelet suppression | 2 | 1 | 27 (90%) |
| Infection | 8 | 10 (34%) | 12 (40%) |
| Nausea/vomiting | 0 | 15 (50%) | 15 (50%) |
| Alopecia | 0 | 0 | 30 (100%) |

There was no significant, neuro-, nephro- or oto- toxicity. This combination is feasible and highly active but causes severe myelosuppression, and would only be of clinical value in LD patients if good long term control can be achieved.

### GR38032F in the control of ifosfamide induced nausea and vomiting

J.A. Green, S.W. Watkin & P. Hammond

*CRC Department of Radiation Oncology, Clatterbridge Hospital, Bebington, Merseyside, UK.*

Ifosfamide (I) is one of the most active drugs in the treatment of non-small cell lung cancer, and is also used in the treatment of small cell lung cancer. One of the principal side effects is prolonged WHO II or III nausea and vomiting over 24–36 h. GR38032F is a potent highly selective antagonist of 5HT3 receptors which is effective against drug induced emesis in animals, and preliminary studies in patients have suggested antiemetic activity against cytotoxic chemotherapy regimens. Six patients receiving ifosfamide at a dose of 6.5–8 g as a 24 h infusion along with mesna 9–12 g over 36 h were entered in the study. Four patients had received no prior chemotherapy. GR38032F was given at a dose of 4 mg 15 min before the start of the ifosfamide infusion and for 6 further doses at 6 hourly intervals. Response was assessed as the number of emetic episodes (any vomit productive of liquid or 1–5 retches within a 5 min period) over the 48 h after the start of I infusion. Two patients had no nausea or vomiting in the study period, 2 patients experienced only one emetic episode, and the 2 patients who had received prior cytotoxic chemotherapy had 4 and 2 emetic episodes respectively. One further patient treated with a dose of 8 mg every 6 h had no nausea, retching or vomiting. No abnormality in haematological or biochemical parameters were detected, and only one patient experienced moderate drowsiness, which was considered to be WHO Grade II CNS toxicity related to the I. It is concluded that GR38032F is a safe and effective antiemetic in the control of ifosfamide induced nausea and vomiting.

### Randomised trial of planned *versus* as required chemotherapy in small cell lung cancer (SCLC)

R. Souhami[1], C. Ash[1], S. Spiro[2], D. Geddes[3], J. Tobias[1], P. Harper[4], H. Earl[1] & L. James[1]

[1]*Department of Clinical Oncology, University College and Middlesex School of Medicine, London, WC1;* [2]*Brompton Hospital, London;* [3]*London Chest Hospital, London; and* [4]*Guy's Hospital, London, UK.*

The poor prognosis of most patients with SCLC has led to assessment of a variety of palliative approaches to chemotherapy. This trial assesses the value of using chemotherapy according to the rate of progression of the disease in the invididual patient.

Patients with both limited or extensive SCLC with poor prognostic features (Souhami *et al., Cancer Res.* **45**, 2878, 1985) have been entered into a randomised trial. In one group chemotherapy (cyclophosphamide 1 gm$^{-2}$ day 1, vincristine 2 mg day 1, etoposide 100 mg bd days 1–3) is given every 3 weeks for a total of 8 cycles. In the other group patients receive the same chemotherapy only when there is disease progression. Patients with responding or stable disease who are asymptomatic are not treated but are seen every 3 weeks and reassessed. 170 patients have been randomised up to May 1987. In the 'as required' arm the median treatment free interval between course 1 and 2 was 47 days; course 2 and 3: 46 days; course 3 and 4: 51 days; course 4 and 5: 46 days. These intervals are over twice as long as the 'planned' chemotherapy. So far there is no difference in survival in the two arms. Quality of life assessments of the two forms of treatment are in progress. This trial represents a new approach to palliative treatment of SCLC.

### Treatment duration in small cell lung cancer (SCLC): A randomised trial

R. Souhami[1], H. Earl[1], C. Ash[1], S. Spiro[2], D. Geddes[3], P. Harper[4], J. Tobias[1] & H. Quinn[1]

[1]*Department of Clinical Oncology, University College and Middlesex School of Medicine, London; WC1,* [2]*Brompton Hospital, London;* [3]*London Chest Hospital, London; and* [4]*Guy's Hospital, London, UK.*

616 patients with both limited and extensive SCLC were entered into a randomised trial comparing two durations of initial treatment and the value of chemotherapy on relapse. Patients were staged by isotope bone scan and liver ultrasound, and stratified according to extent (limited or or extensive). Patients were then randomised to receive either 4 or 8 courses of chemotherapy (cyclophosphamide 1 gm$^{-2}$ day 1, vincristine 2 mg day 1, etoposide 100 mg tds days 1–3) 3 weekly. At presentation patients were also randomised for treatment at relapse, either to receive further chemotherapy (doxorubicin 50 mg m$^{-2}$, and methotrexate 50 mg m$^{-2}$, every 3 weeks) or symptomatic treatment alone. 610 patients were evaluable for response and survival.

Response to short (S) and long (L) initial chemotherapy were similar (S=61%, L=63%), as were response rates to relapse chemotherapy (S=25%, L=18%). Overall median survival (MS) from course 1 was analyised by intention to treat, and patients randomised to 8 courses of initial chemotherapy had a slightly longer MS than those randomised to 4 courses (MS, 39 *vs.* 32 weeks, $P=0.085$). Progression free interval (PFI) after initial chemotherapy was longer in patients receiving 8 rather than 4 courses (Median, 31 *vs.* 23 weeks, $P=0.0002$). Survival from relapse was longer for patients receiving relapse chemotherapy than for those receiving symptomatic treatment alone (MS, 17 *vs.* 12 weeks, $P=0.0004$). Patients responding to chemotherapy had a shorter relapse free interval with 4 cycles, and a worse overall survival (MS, 42 *vs.* 52 weeks, $P=0.02$). This difference was not apparent if chemotherapy was given on relapse. Short initial chemotherapy therefore gives worse survival than long, unless relapse chemotherapy is given.

### Surgical resection and adjuvant chemotherapy for limited stage small cell carcinoma (SCLC)

S.W. Watkin[1], D. Kaplan[2], J.A. Green[1] & R.J. Donnelly[2]

[1]*University of Liverpool Department of Radiation Oncology, Clatterbridge Hospital, Bebington, Wirral, Merseyside, L63 4JY; and* [2]*Regional Adult Cardiothoracic Unit, Broadgreen Hospital, Thomas Drive, Liverpool 14, UK.*

Between 1980–1986 33 patients, 21 male and 12 female, aged 41–72 years, mean 60.3 years underwent surgery for SCLC. There were 15 lobectomies and 18 pneumonectomies. Preoperative diagnosis was SCLC in 13 (39%), NSCLC in 3 (9%) and in 6 the pre-operative biopsy was non-diagnostic. Of the remainder biopsy was not possible in 6 (18%) and there was insufficient data in 5 (15%). Post-operative histology was SCLC in all cases and in one there were elements of adenocarcinoma. Disease was confined to the lung in 17 cases and at thoracotomy 16 were found to be locally advanced or have residual microscopic disease. Seventeen patients received adjuvant chemotherapy and 3 also received prophylactic cranial irradiation. 8/17 received chemotherapy with 4 cycles of a combination chemotherapy regime (CAV+IMVP-16). Seven patients are alive at 6–53 months of whom 4 have not relapsed and there have been 17 deaths, none of which were related to chemotherapy. There were 3 early post-operative deaths and there is incomplete follow-up in 9. Median survival of the 24 patients with complete data is 14 months, 1 year survival 55% and 2 year survival 22%. These results suggest that surgery is an effective form of treatment for selected patients with limited stage SCLC when combined with intensive adjuvant chemotherapy.

### High dose chemotherapy with autologous bone marrow rescue in small cell lung cancer

C. Wilson, D. Pickering, S. Stewart, K. Vallis, H. Kalofonos, A. Cross, D. Snook, J. Goldman, C.G. McKenzie & A. A. Epenetos

*Departments of Clinical Oncology and Haematology, Hammersmith Hospital, London, UK.*

Twenty five consecutive patients diagnosed as having small cell lung cancer (SCLC) were treated with etoposide and *cis*-platinum. After assessment for tumour response, autologous bone marrow (ABM) was collected from 5 patients in good clinical state who had shown a partial response (PR) and no marrow involvement with tumour. These patients were treated with high dose *cis*-platinum, etoposide and melphalan and then by ABM infusion. Although all patients (3 with extensive and 2 with limited disease) achieved complete remission (CR), 3 patients with extensive disease relapsed and died of their disease. One patient with limited disease died of infection while pancytopenic; postmortem examination in this patient showed the presence of tumour. One patient who achieved CR, received local chest irradiation and continues in remission. Although patient numbers are small, our data support the conclusion that high dose chemotherapy and autologous bone marrow rescue has a very small role if any, in the management of patients with extensive or limited stage SCLC.

This conference was organised by the Clatterbridge Cancer Research Trust. We thank Beecham Research, Boehringer Ingelheim and Bristol Myers Oncology for sponsorship; and Blaxo, Roche and Cancer Research Campaign for additional financial contributions.